# Accelerating Sustainable Development Goals for South African adolescents from high HIV prevalence areas: a longitudinal path analysis

**DOI:** 10.1186/s12916-021-02137-8

**Published:** 2021-11-11

**Authors:** Franziska Meinck, Mark Orkin, Lucie Cluver

**Affiliations:** 1 School of Social and Political Sciences, University of Edinburgh, 15a George Square, Edinburgh, EH8 9LD UK; 2 OPTENTIA, Faculty of Humanities, North-West University, Vanderbijlpark, South Africa; 3 School of Public Health, University of the Witwatersrand, Johannesburg, South Africa; 4 MRC-Wits Developmental Pathways for Health Research Unit, School of Clinical Medicine, University of the Witwatersrand, Johannesburg, South Africa; 5 Centre for Evidence-Based Intervention, Department of Social Policy and Intervention, University of Oxford, Oxford, UK; 6 Department of Psychiatry and Mental Health, University of Cape Town, Cape Town, South Africa

**Keywords:** Accelerators, Sustainable Development Goals, Violence prevention, Adolescents, Parenting, Food sufficiency, HIV/AIDS, Child abuse, Social protection, Mental health

## Abstract

**Background:**

Adolescents experience a multitude of vulnerabilities which need to be addressed in order to achieve the Sustainable Development Goals (SDGs). In sub-Saharan Africa, adolescents experience high burden of HIV, violence exposure, poverty, and poor mental and physical health. This study aimed to identify interventions and circumstances associated with three or more targets (“accelerators”) within multiple SDGs relating to HIV-affected adolescents and examine cumulative effects on outcomes.

**Methods:**

Prospective longitudinal data from 3401 adolescents from randomly selected census enumeration areas in two provinces with > 30% HIV prevalence carried out in 2010/11 and 2011/12 were used to examine six hypothesized accelerators (positive parenting, parental monitoring, free schooling, teacher support, food sufficiency and HIV-negative/asymptomatic caregiver) targeting twelve outcomes across four SDGs, using a multivariate (multiple outcome) path model with correlated outcomes controlling for outcome at baseline and socio-demographics. The study corrected for multiple-hypothesis testing and tested measurement invariance across sex. Percentage predicted probabilities of occurrence of the outcome in the presence of the significant accelerators were also calculated.

**Results:**

Sample mean age was 13.7 years at baseline, 56.6% were female. Positive parenting, parental monitoring, food sufficiency and AIDS-free caregiver were variously associated with reductions on ten outcomes. The model was gender invariant. AIDS-free caregiver was associated with the largest reductions. Combinations of accelerators resulted in a percentage reduction of risk of up to 40%.

**Conclusion:**

Positive parenting, parental monitoring, food sufficiency and AIDS-free caregivers by themselves and in combination improve adolescent outcomes across ten SDG targets. These could translate to the corresponding real-world interventions parenting programmes, cash transfers and universal access to antiretroviral treatment, which when provided together, may help governments in sub-Saharan Africa more economically to reach their SDG targets.

**Supplementary Information:**

The online version contains supplementary material available at 10.1186/s12916-021-02137-8.

## Background

Adolescents make up 23% of Africa’s population, and Africa’s adolescents are the fastest growing population group in the world [1]. However, they experience a multitude of vulnerabilities which often go unaddressed. These include increased risk for loss of a parent or illness of a parent due to AIDS and the associated vulnerabilities [2], HIV infection among girls [3] as well as exposure to violence [4], early child bearing [5], poor mental health [6], and challenges in accessing and remaining in education [7]. Urgent intervention at the government and policy level is needed to attain the Sustainable Development Goals (SDGs) in this region and ensure adolescents reach their full potential.

Reaching all the SDG targets in the nine years that remain until 2030 is a major challenge for all governments. Development accelerators, promoted by UNDP, are considered to be a new approach which could help governments deliver across targets and goals [8]. UNDP define accelerators as “pragmatic actions”—in practice, interventions such as parenting programmes, or circumstances such as adequate food provision—that have a beneficial association with multiple SDG outcomes [9]. Accelerators are therefore provisions, protective factors or interventions which reduce poor outcomes for adolescents on at least three sustainable SDG targets. For example, a recent study from South Africa with HIV-positive adolescents showed that supportive parenting was associated with good mental health and no violence perpetration, no high-risk sex, no community violence exposure nor physical or emotional child abuse (SDGs 3.3, 3.4, 5.2, 16.2, 16.1 and 16.2 respectively )[10]. Therefore, supportive parenting was considered an accelerator as it was associated with reductions across five SDG targets. In addition, this study showed substantial additive effects where different accelerators together were associated with significant improvements on a particular outcome. However, studies have been lacking that investigate accelerators of adolescents in contexts with high parental HIV prevalence, which has been shown to be associated with poor child health outcomes and other vulnerabilities [11].

The present study therefore has four aims: (1) to investigate the association of six pragmatic actions with twelve SDG target outcomes and to identify any accelerators among these pragmatic actions in a sample of South African adolescents in areas with high HIV prevalence; (2) to take account of correlations among the outcomes as necessary; (3) to check for moderation of associations by respondents’ sex; and (4) to test additive effects of combined accelerators on SDG target outcomes.

## Methods

### Procedure

We consulted prior to baseline with local traditional leaders, local provincial government, pre-piloted with adolescents from these population groups, and introduced the study at multiple community meetings. The design of the study and questionnaire was carried out in consultation with our South African Teen Advisory Group [12].

### Participants

Adolescents (*n* = 3516) aged 10–17 (56.7% female) were recruited from urban and rural areas in two South African provinces, interviewed at baseline (2010–2011) and followed up 1 year later. Refusal rate at baseline was < 2.5%, and retention rate at one-year follow-up was 96.8%. Door-to-door sampling was used to recruit adolescents in randomly selected census enumeration areas across four health districts with antenatal HIV prevalence > 30%. Areas were highly deprived and previously disadvantaged homelands or townships. In 70-min face-to-face interviews, one randomly selected adolescent was interviewed per household, by interviewers trained in working with vulnerable youth. No exclusion criteria were applied unless the adolescent was deemed incapable of giving consent or understanding the questionnaire. Informed consent was sought from the primary caregiver and assent from the adolescent. Consent forms and questionnaires were translated into the six local languages and checked with back translation.

Ethical clearance was provided by the provincial government departments of Health and Education, the Universities of Oxford (SSD/CUREC2/09-52), Cape Town (389/209) and KwaZulu-Natal (HSS/0254/09). Participants were interviewed in private spaces such as gardens to ensure privacy and confidentiality. Confidentiality was maintained throughout the study except where participants requested help or were at risk of significant harm, where referrals, in line with mandatory reporting requirements, were made to health or statutory child protective services with follow-up support. We supported children to disclose to their caregivers where these were not the cause of harm. All participants received a certificate and refreshments.

## Measures

### SDG outcomes

SDG outcomes were binary. SDG 16.2 *Physical abuse* in the past year was measured using two items from the UNICEF Scales for National-Level Monitoring of Orphans and Other Vulnerable Children [13]. SDG 16.2 *Emotional abuse* in the past year was measured using three items from the same measure. SDG 5.2 *Sexual abuse* was measured using two items from the Juvenile Victimization Questionnaire [14]. Items were dichotomized into past-year abuse experience vs none. SDG 4.A *Bullying* (*α* = .81) was measured using the 8-item Social and Health Assessment Peer Victimization Scale [15]. A cut-off of 9 was used to define experience of bullying vs no bullying. SDG 5.2 *Witnessing domestic violence* was measured using one item as one or more incidents in which adults were hitting or shouting at each other violently in the home in the past week [13]. SDG 16.1 *Exposure to Community Violence* in the form of ever being robbed or assaulted was measured using two items from the Child Exposure to Community Violence (CECV) Checklist [16] and defined as one or more incidents. SDG 16.1 *Experience of Community Violence* through ever witnessing a shooting or stabbing was also measured using two binary items from the CECV and defined as occurrence vs no occurrence. SDG 3.3 *TB symptomology* was assessed using an 8-item TB symptom checklist with two or more symptoms of chest pains, coughing blood and cough for three weeks defined as TB symptomatic taken from the KwaZulu-Natal Department of Health TB symptom guidelines. SDG 3.4 *Suicidal ideation and attempts* during the past month were measured using the Mini International Psychiatric Interview for Children and Adolescents (MINI-KID) [17] which has been previously used in South Africa and showed acceptable internal consistency in this sample (*α* = .73) and defined as 1+ endorsements of five items. SDG 5.61 *HIV risk behaviour* was measured using three binary items from the National Survey of HIV and Sexual Behaviour among Young South Africans [18] measuring sex before age 15, having sex drunk or on drugs, and transactional sex and one item from the South African Demographic and Health Survey [19] on sex with partners 5+ years older, and was defined as engaging in one or more of these behaviours. SDG 3.5 *Alcohol and drug use* in the past month was measured using two items from the National Survey of HIV and Risk Behaviours among Young South Africans [18] measuring alcohol use and use of seven common substances and defined as using one or more of these substances. SDG 4.1 *School Dropout* was measured as child no longer enrolled in school.

### Hypothesized accelerators

All hypothesized accelerators were required to have been provided at both baseline and follow-up and were binary. An accelerator was defined as an intervention or circumstance that has a beneficial association with three or more SDG targets as calculated through the study analysis [9]. *Positive Parenting* was measured using the 4-item subscale from the Alabama Parenting Questionnaire Short Form (APQ-SF )[20], and defined as children reporting receipt of positive parenting always or mostly on all items. *Parental Monitoring* was measured using the 3-item subscale from the APQ-SF and defined as children reporting receipt of good monitoring always or mostly on all items. *Food sufficiency* was measured as child reporting neither going to bed hungry nor being hungry at school in the past 7 days. *AIDS-free caregiver* was measured using Verbal Autopsy methods validated in previous studies of adult mortality in South Africa [21]. The Verbal Autopsy is an 18-item checklist of current common health conditions and AIDS-defining illnesses. Determination of caregiver HIV/AIDS-symptomology required endorsement of three or more AIDS-defining illnesses (i.e. tuberculosis, shingles or Kaposi’s sarcoma). *Teacher emotional support* was measured using the 2 items from the Social Support Scale and defined as having a teacher who provides very good emotional or instrumental support [22]. *Free schooling* was defined as attending a *no-fees school*, receiving a *free school meal* and *free textbooks* at the same time at both baseline and follow-up. Attendance at *no-fees schools* was almost universal but provision of *free school meals* and *free textbooks* varied by school independent of parental income. All measures have been previously used in South Africa.

### Pre-selected covariates

Hypothesized accelerators at *baseline* were measured with the above items. *Urban/rural location, province, informal housing, sex* and *age* were measured with categories from the South African census [23].

## Analysis

**Fig. 1:**
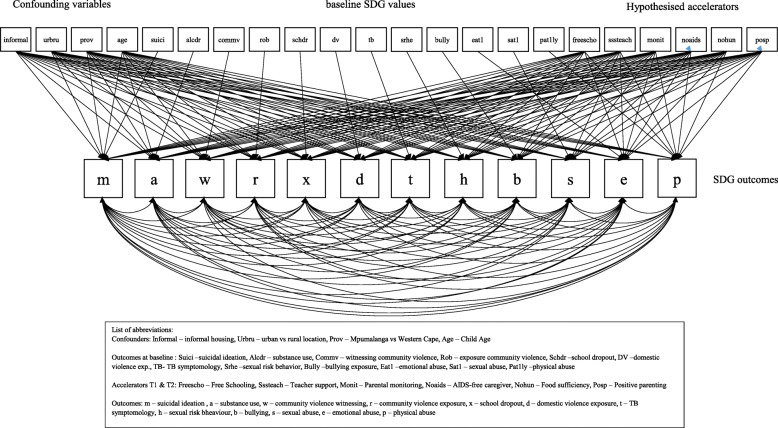
Hypothesized accelerator model for adolescent SDG outcomes with correlated outcomes

Analyses were conducted in seven stages using Stata 15 and Mplus 8.3 (all syntax can be found in Additional File 2). First, descriptive statistics were calculated for all hypothesized accelerators, SDG outcomes and covariates (Table 1) in Stata 15.1. Second, associations between all variables were checked in a correlation matrix (Additional File 1: Supplement 1) to assess if correlation of outcomes would be required [24]. Third, multivariate (multiple-outcome) probit regressions were conducted in MPlus 8.0 to identify potential accelerators, by entering all chosen SDG outcomes and hypothesized accelerator variable and covariates (baseline exposure, age, province, urban location, informal housing and sex) simultaneously as a path model (Table 2). The model included the Mplus command for correlation among all outcomes (Fig. 1). Standardized correlation coefficients for SDG outcomes are reported in Supplement 2 (Additional File 1). Fourth, to check for possible moderation of paths by participant sex, path invariance was tested between the configural model and the model with all paths constrained to equality across the two groups, using the Mplus DIFFTEST command. Fifth, to account for multiple-hypothesis testing and risk of type I error, the Benjamini-Hochberg procedure was used with a specified false discovery rate of 0.05 [25]. Sixth, all predictors which were positively associated with at least three SDG outcomes were defined as accelerators. Finally, predicted probabilities were calculated for instances where two or more accelerators were associated with a given outcome, holding covariates at their mean value, and probabilities were extracted for different summative combinations of accelerators on SDG outcomes (Table 3). These analyses in MPlus were conducted using weighted least squares and variance adjusted (WLSMV) estimation, due to categorical data for the SDG outcomes. Model fit was assessed using *χ*^2^/df, comparative fit index (CFI), Tucker-Lewis Index (TLI), root mean square error of approximation (RMSEA) and weighted root mean square residual (WRMR). By convention, the maximum acceptable value for *χ*^2^/df is 5. A value of > .95 indicates good fit for CFI and TLI, < .05 for RMSEA and > 0.95 for WRMR [26]. Missing data were less than 1% on all variables and thus no imputation was conducted; but pairwise deletion was used as the standard with the WLSMV estimator.

**Table 1:** Sociodemographic characteristics of the study sample (*N* = 3401)

	Total *n* = 3401	Boys *n* = 1475 (43.4%)	Girls *n* = 1926 (56.6%)	Sex difference *p* value
**Sociodemographic characteristics at baseline**				
Province				0.030
Western Cape	1753 (51.5%)	729 (41.6%)	1024 (58.4%)	
Mpumalanga	1648 (48.5%)	746 (45.3%)	902 (54.7%)	
Age				0.615
Mean (SD)	13.43 (2.14)	13.4 (2.10)	13.44 (2.18)	
Rural location				0.238
Yes	1681 (49.4%)	712 (42.4%)	969 (57.6%)	
Informal housing				0.153
Yes	1068 (31.4%)	444 (41.6%)	624 (58.4)	
**Hypothesized protective factors received at both T1 and T2**				
Positive parenting				0.502
Yes	875 (25.7%)	371 (42.4%)	504 (57.6%)	
Parental monitoring				< 0.001
Yes	2677 (78.7%)	1105 (41.2%)	1572 (58.7%)	
Food sufficiency at home				0.776
Yes	2829 (83.2%)	1230 (43.48)	1599 (56.5%)	
HIV−/ asymptomatic caregiver				0.057
Yes	3198 (94.0%)	1400 (43.8%)	1798 (56.2%)	
Teacher support				0.365
Yes	569 (16.7%)	237 (41.7%)	332 (58.4%)	
Free school meals, textbooks and no fees				0.243
Yes	1329 (39.1%)	560 (42.1%)	769 (57.8%)	
**SDG outcomes at T2**				
Physical abuse				0.226
Yes	1289 (37.9%)	576 (44.7%)	713 (55.31)	
Emotional abuse				0.004
Yes	1076 (31.6%)	428 (39.8%)	648 (60.2%)	
Sexual abuse				0.001
Yes	295 (8.7%)	101 (34.2%)	194 (65.8%)	
Bullying				0.022
Yes	2494 (73.3%)	1111 (44.6%)	1383 (55.5%)	
Domestic violence witnessing				0.002
Yes	535 (15.7%)	200 (37.4%)	335 (62.6%)	
Community violence exposure				0.723
Yes	1312 (38.6%)	574 (43.8%)	738 (56.3%)	
Community violence witnessing				0.124
Yes	1338 (39.3%)	602 (45.0%)	736 (55.0%)	
Suicidal ideation				< 0.001
Yes	614 (18.1%)	213 (34.7%)	401 (65.7%)	
HIV risk behaviour				< 0.001
Yes	443 (13.0%)	251 (56.7%)	192 (43.3%)	
TB symptomatic				0.105
Yes	176 (5.2%)	66 (37.5%)	110 (62.5%)	
Substance use				< 0.001
Yes	249 (7.3%)	143 (57.4%)	106 (42.6%)	
School dropout				0.046
Yes	147 (4.3%)	52 (35.4%)	95 (64.6%)

**Table 2:** Path model using multivariate probit regressions with correlated outcomes reporting standardized coefficients using the WLSMV estimator (*n* = 3396)

	Coefficient	95% CI	S.E.	*p* value
**Physical abuse**				
Positive parenting	− 0.066	− 0.11 to − 0.022	0.022	0.003
Food sufficiency	− 0.056	− 0.098 to − 0.013	0.022	0.010
AIDS-free caregiver	− 0.107	− 0.147 to − 0.067	0.020	0.000
Parental monitoring	− 0.083	− 0.126 to − 0.039	0.022	0.000
Teacher support	− 0.013	− 0.057–0.03	0.022	0.546
Free schooling	0.048	0.002–0.093	0.023	0.043
Baseline physical abuse	0.079	0.033–0.125	0.023	0.001
Age	− 0.154	− 0.2 to − 0.108	0.023	0.000
Province	0.230	0.168–0.292	0.032	0.000
Urban location	0.057	0.011–0.103	0.023	0.015
Informal housing	− 0.008	− 0.061–0.044	0.027	0.758
Sex	− 0.011	− 0.053–0.032	0.022	0.625
**Emotional abuse**				
Positive parenting	− 0.074	− 0.12 to − 0.027	0.024	0.002
Food sufficiency	− 0.095	− 0.138 to − 0.052	0.022	0.000
AIDS-free caregiver	− 0.127	− 0.168 to − 0.085	0.021	0.000
Parental monitoring	− 0.085	− 0.129 to − 0.04	0.023	0.000
Teacher support	0.038	− 0.006–0.082	0.023	0.091
Free schooling	0.020	− 0.027–0.068	0.024	0.405
Baseline emotional abuse	0.092	0.044–0.141	0.025	0.000
Age	0.008	− 0.041–0.056	0.025	0.758
Province	− 0.007	− 0.072–0.059	0.033	0.844
Urban location	0.028	− 0.019–0.076	0.024	0.247
Informal housing	− 0.050	− 0.103–0.003	0.027	0.066
Sex	0.059	0.014–0.103	0.023	0.010
**Sexual abuse**				
Positive parenting	− 0.064	− 0.131–0.002	0.034	0.058
Food sufficiency	− 0.046	− 0.108–0.015	0.031	0.141
AIDS-free caregiver	− 0.045	− 0.101–0.012	0.029	0.122
Parental monitoring	− 0.040	− 0.101–0.02	0.031	0.190
Teacher support	0.009	− 0.054–0.072	0.032	0.772
Free schooling	− 0.002	− 0.068–0.065	0.034	0.961
Baseline sexual abuse	0.135	0.091–0.179	0.023	0.000
Age	0.138	0.07–0.206	0.035	0.000
Province	0.211	0.122–0.299	0.045	0.000
Urban location	0.028	− 0.039–0.096	0.034	0.411
Informal housing	− 0.097	− 0.177 to − 0.017	0.041	0.018
Sex	0.105	0.042–0.167	0.032	0.001
**Witnessing domestic violence**				
Positive parenting	− 0.027	− 0.081–0.028	0.028	0.335
Food sufficiency	− 0.112	− 0.16 to − 0.064	0.025	0.000
AIDS-free caregiver	− 0.067	− 0.114 to − 0.021	0.024	0.004
Parental monitoring	0.000	− 0.054–0.055	0.028	0.993
Teacher support	− 0.032	− 0.086–0.022	0.028	0.244
Free schooling	− 0.025	− 0.083–0.032	0.029	0.392
Baseline domestic violence	0.112	0.059–0.165	0.027	0.000
Age	− 0.043	− 0.098–0.011	0.028	0.121
Province	− 0.114	− 0.189 to − 0.039	0.038	0.003
Urban location	− 0.113	− 0.167 to − 0.058	0.028	0.000
Informal housing	− 0.022	− 0.081–0.038	0.030	0.472
Sex	0.067	0.015–0.12	0.027	0.012
**Bullying**				
Positive parenting	0.030	− 0.017–0.078	0.024	0.213
Food sufficiency	− 0.077	− 0.124 to − 0.031	0.024	0.001
AIDS-free caregiver	− 0.072	− 0.121 to − 0.023	0.025	0.004
Parental monitoring	− 0.069	− 0.116 to − 0.022	0.024	0.004
Teacher support	− 0.043	− 0.087–0.002	0.023	0.062
Free schooling	0.042	− 0.007–0.092	0.025	0.091
Baseline bullying	0.128	0.083–0.173	0.023	0.000
Age	− 0.109	− 0.156 to − 0.061	0.024	0.000
Province	− 0.013	− 0.081–0.055	0.035	0.711
Urban location	− 0.158	− 0.206 to − 0.111	0.024	0.000
Informal housing	0.034	− 0.021–0.09	0.028	0.224
Sex	− 0.045	− 0.091–0.001	0.023	0.053
**Witnessing community violence**				
Positive parenting	− 0.030	− 0.071–0.01	0.021	0.145
Food sufficiency	− 0.059	− 0.095 to − 0.023	0.018	0.001
AIDS-free caregiver	− 0.021	− 0.061–0.019	0.020	0.299
Parental monitoring	− 0.035	− 0.073–0.003	0.020	0.073
Teacher support	0.038	− 0.002–0.077	0.020	0.063
Free schooling	0.001	− 0.039–0.041	0.021	0.954
Baseline wit. community violence	0.148	0.11–0.186	0.020	0.000
Age	0.108	0.068–0.148	0.021	0.000
Province	− 0.539	− 0.584 to − 0.493	0.023	0.000
Urban location	− 0.054	− 0.094 to − 0.013	0.021	0.009
Informal housing	− 0.025	− 0.068–0.018	0.022	0.261
Sex	− 0.051	− 0.09 to − 0.013	0.020	0.009
**Experiencing community violence**				
Positive parenting	− 0.030	− 0.072–0.013	0.022	0.172
Food sufficiency	− 0.065	− 0.104 to − 0.026	0.020	0.001
AIDS-free caregiver	− 0.084	− 0.126 to − 0.043	0.021	0.000
Parental monitoring	− 0.046	− 0.087 to − 0.005	0.021	0.029
Teacher support	0.040	− 0.001–0.08	0.021	0.055
Free schooling	0.024	− 0.02–0.068	0.023	0.288
Baseline Exp Comm Viol	0.057	0.015–0.1	0.022	0.008
Age	0.039	− 0.005–0.083	0.022	0.080
Province	− 0.414	− 0.467 to − 0.362	0.027	0.000
Urban location	− 0.291	− 0.332 to − 0.25	0.021	0.000
Informal housing	− 0.063	− 0.111 to − 0.016	0.024	0.008
Sex	− 0.022	− 0.063–0.018	0.021	0.282
**School dropout**				
Positive parenting	− 0.046	− 4.538–4.446	2.292	0.984
Food sufficiency	− 0.080	− 7.944–7.784	4.012	0.984
AIDS-free caregiver	0.008	− 0.827–0.844	0.426	0.984
Parental monitoring	− 0.020	− 1.941–1.902	0.980	0.984
Teacher support	− 0.378	− 37.417–36.661	18.897	0.984
Free schooling	− 0.026	− 2745.612–2745.559	1400.809	1.000
Baseline school dropout	0.146	− 14.144–14.435	7.291	0.984
Age	0.307	− 29.8–30.415	15.361	0.984
Province	0.001	− 0.188–0.191	0.097	0.989
Urban location	0.091	− 8.844–9.026	4.559	0.984
Informal housing	0.049	− 4.74–4.838	2.443	0.984
Sex	0.066	− 6.426–6.559	3.312	0.984
**Substance use**				
Positive parenting	− 0.111	− 0.192 to − 0.029	0.042	0.008
Food sufficiency	− 0.023	− 0.084–0.037	0.031	0.450
AIDS-free caregiver	− 0.031	− 0.093–0.032	0.032	0.339
Parental monitoring	− 0.088	− 0.147 to − 0.028	0.030	0.004
Teacher support	− 0.003	− 0.071–0.065	0.035	0.930
Free schooling	− 0.056	− 0.127–0.016	0.037	0.129
Baseline substance use	0.044	− 0.02–0.108	0.033	0.180
Age	0.321	0.257–0.384	0.032	0.000
Province	− 0.258	− 0.353 to − 0.164	0.048	0.000
Urban location	− 0.009	− 0.075–0.057	0.034	0.790
Informal housing	− 0.080	− 0.147 to − 0.012	0.034	0.020
Sex	− 0.135	− 0.201 to − 0.069	0.034	0.000
**Suicidal ideation**				
Positive parenting	− 0.059	− 0.114 to − 0.005	0.028	0.033
Food sufficiency	− 0.097	− 0.143 to − 0.051	0.023	0.000
AIDS-free caregiver	− 0.104	− 0.148 to − 0.061	0.022	0.000
Parental monitoring	0.002	− 0.047–0.052	0.025	0.923
Teacher support	− 0.048	− 0.101–0.005	0.027	0.075
Free schooling	− 0.024	− 0.078–0.03	0.028	0.387
Baseline suicidal ideation	0.140	0.095–0.186	0.023	0.000
Age	0.121	0.07–0.173	0.026	0.000
Province	− 0.102	− 0.175 to − 0.029	0.037	0.006
Urban location	− 0.139	− 0.19 to − 0.087	0.026	0.000
Informal housing	− 0.032	− 0.09–0.026	0.030	0.283
Sex	0.095	0.044–0.146	0.026	0.000
**HIV risk behaviour**				
Positive parenting	− 0.006	− 0.065–0.054	0.030	0.854
Food sufficiency	− 0.060	− 0.11 to − 0.011	0.025	0.017
AIDS-free caregiver	− 0.068	− 0.116 to − 0.02	0.025	0.006
Parental monitoring	− 0.072	− 0.12 to − 0.024	0.025	0.003
Teacher support	0.008	− 0.047–0.063	0.028	0.776
Free schooling	− 0.087	− 0.144 to − 0.03	0.029	0.003
Baseline HIV risk behaviour	0.193	0.155–0.232	0.020	0.000
Age	0.326	0.27–0.383	0.029	0.000
Province	− 0.162	− 0.235 to − 0.088	0.038	0.000
Urban location	0.026	− 0.029–0.081	0.028	0.360
Informal housing	0.004	− 0.054–0.062	0.029	0.891
Sex	− 0.166	− 0.219 to − 0.114	0.027	0.000
**TB symptomatic**				
Positive parenting	− 0.029	− 0.107–0.049	0.040	0.465
Food sufficiency	0.065	− 0.017–0.148	0.042	0.122
AIDS-free caregiver	− 0.088	− 0.149 to − 0.026	0.031	0.005
Parental monitoring	− 0.013	− 0.096–0.07	0.042	0.763
Teacher support	0.009	− 0.066–0.083	0.038	0.821
Free schooling	0.040	− 0.044–0.123	0.043	0.352
Baseline TB symptomology	0.135	0.075–0.196	0.031	0.000
Age	− 0.012	− 0.097–0.073	0.043	0.780
Province	0.224	0.115–0.333	0.056	0.000
Urban location	− 0.067	− 0.154–0.02	0.044	0.129
Informal housing	0.016	− 0.085–0.116	0.051	0.760
Sex	0.057	− 0.019–0.132	0.038	0.140

**Model fit:** *χ*^*2*^ = 258.387, df = 132, *p* < 001, CFI .967, TLI .914, RMSEA .017, WRMR .878

**Table 3.** Adjusted predicted percentage probabilities for experiencing SDG outcomes and no, one, and all significant accelerators

	Percentage probability	Confidence interval	Difference in % probability compared to no accelerator
**Physical abuse**			
No accelerator	57.2	47.2–67.1	
Positive parenting	50.9	40.2–61.5	6.1 (2.3–9.9)
Food sufficiency	50.9	41.6–60.3	6.1 (1.6–10.6)
AIDS-free caregiver	38.4	31.3–45.5	18.6 (11.6–25.6)
Parental monitoring	48.7	39.4–58.0	8.3 (4.2–12.4)
Positive parenting & food sufficiency	44.6	34.8–54.3	12.6 (8.6–16.6)
Positive parenting & AIDS-free caregiver	32.5	25.2–39.8	24.7 (20.9–28.5)
Positive parenting & parental monitoring	42.4	32.8–51.9	14.8 (10.8–18.8)
Food sufficiency & AIDS-free caregiver	32.6	26.8–38.3	24.6 (20.7–28.5)
Food sufficiency & parental monitoring	42.4	34–50.8	14.8 (10.8–18.8)
AIDS-free caregiver & parental monitoring	30.6	25.0–36.1	21.2 (17.5–24.9)
Positive parenting, food sufficiency & AIDS-free caregiver	27.0	21.2–32.8	30.2 (26.2–34.2)
Positive parenting, food sufficiency & parental monitoring	36.3	27.9–44.7	21.2 (17.0–25.4)
Positive parenting, AIDS-free caregiver & parental monitoring	25.2	19.7–30.7	32.0 (28.0–36.0)
Food sufficiency, AIDS-free caregiver & parental monitoring	25.3	21.1–29.4	31.9 (28.7–35.1)
Positive parenting, food sufficiency, AIDS-free caregiver & parental monitoring	20.4	16.4–24.5	32.2 (29.0–37.4)
**Emotional abuse**			
No accelerator	53.0	42.8–63.1	
Positive parenting	46.1	35.4–56.7	6.9 (3.1–10.7)
Food sufficiency	42.6	33.2–51.9	10.4 (5.9–14.9)
AIDS-free caregiver	31.7	25.2–38.2	21.3 (14.2–28.4)
Parental monitoring	44.5	35.2–53.7	8.5 (4.4–12.6)
Positive parenting & food sufficiency	35.9	26.6–45.3	17.1 (13.1–21.1)
Positive parenting & AIDS-free caregiver	25.8	19.4–32.2	27.2 (23.4–31.0)
Positive parenting & parental monitoring	37.7	28.4–47.1	15.3 (11.3–19.3)
Food sufficiency & AIDS-free caregiver	23.0	18.2–27.9	30.0 (26.4–33.6)
Food sufficiency & parental monitoring	34.5	26.4–42.5	18.5 (15.1–21.9)
AIDS-free caregiver & parental monitoring	24.5	19.7–29.4	28.5 (25.1–31.9)
Positive parenting, food sufficiency & AIDS-free caregiver	18.1	13.5–22.7	34.9 (31.0–38.9)
Positive parenting, food sufficiency &parental monitoring	28.3	20.6–36.1	24.7 (20.6–28.8)
Positive parenting, AIDS-free caregiver & parental monitoring	19.4	14.8–24.1	33.6 (29.7–37.5)
Food sufficiency, AIDS-free caregiver & parental monitoring	17.1	13.8–20.4	35.9 (32.9–38.9)
Positive parenting, food sufficiency, AIDS-free caregiver & parental monitoring	13.0	10.0–16.1	40.0 (35.9–44.1)
**Bullying**			
No accelerator	85.7	79.4–91.9	
Food sufficiency	80.2	72.9–87.5	5.5 (2.3–8.7)
AIDS-free caregiver	77.4	72.1–82.6	8.3 (3.3–13.3)
Parental monitoring	81.3	74.4–88.2	4.4 (1.3–7.5)
Food sufficiency & AIDS-free caregiver	70.4	65.2–75.5	15.3 (11.8–18.8)
Food sufficiency & parental monitoring	75.0	67.4–82.6	10.7 (7.8–13.6)
AIDS-free caregiver & parental monitoring	71.7	66.8–76.6	14.0 (10.7–17.3)
Food sufficiency, AIDS-free caregiver & parental monitoring	64.0	59.8–68.3	21.7 (18.7–24.7)
**HIV risk behaviour**			
No accelerator	22.0	11.8–32.2	
Food sufficiency	16.7	8.8–24.7	5.3 (1.9–8.7)
AIDS-free caregiver	13.3	8.0–18.6	8.7 (3.8–13.6)
Parental monitoring	16.3	8.2–24.4	5.7 (2.6–8.8)
Food sufficiency & AIDS-free caregiver	9.6	5.8–13.3	12.4 (9.8–15.0)
Food sufficiency & parental monitoring	12.0	6.1–17.9	10.0 (7.5–12.5)
AIDS-free caregiver & parental monitoring	9.3	5.6–12.9	12.7 (10.2–15.2)
Food sufficiency, AIDS-free caregiver & parental monitoring	6.5	4.1–8.9	15.5 (13.3–17.7)
**TB symptomatic**			
No accelerator	5.8	1.2–10.4	
AIDS-free caregiver	2.5	0.6–4.4	3.3 (2.7–3.9)
**Witnessing domestic violence**			
No accelerators	27.1	17.6–36.6	
Food sufficiency	17.9	10.7–25.1	9.2 (5.7–12.7)
AIDS-free caregiver	18.3	12.6–24.1	8.8 (3.3–14.3)
Food sufficiency & AIDS-free caregiver	11.3	7.5–15.0	15.8 (13.0–18.6)
**Experiencing community violence**			
No accelerators	55.7	45.0–66.4	
Food sufficiency	47.6	37.4–57.8	8.1 (3.6–12.6)
AIDS-free caregiver	39.3	32.2–46.4	16.4 (9.5–23.3)
Parental monitoring	50.5	40.5–60.5	5.2 (1.1–9.3)
Food sufficiency & AIDS-free caregiver	31.7	25.9–37.5	24.0 (20.2–27.8)
Food sufficiency & parental monitoring	42.4	33.1–51.7	13.3 (9.8–16.8)
AIDS-free caregiver & parental monitoring	34.4	28.5–40.2	21.3 (17.6 –25.0)
Food sufficiency, AIDS-free caregiver & parental monitoring	27.2	22.7–31.7	28.5 (25.3–31.7)
**Witnessing community violence**			
No accelerators	43.3	31.9–54.7	
Food sufficiency	35.3	25–45.5	8.0 (3.7–12.3)
**Substance use**			
No accelerators	7.5	1.5–13.6	
Positive parenting	4.2	0.2–8.1	3.3 (1.4–5.2)
Parental monitoring	4.6	0.8–8.4	2.9 (1.1–4.7)
Positive parenting & parental monitoring	2.4	0.0–4.7	5.1 (3.1–7.1)
**Suicide ideation**			
No accelerators	32.4	22.6–42.3	
Positive parenting	27.4	17.9–37	5.0 (1.4–8.6)
Food sufficiency	23.2	15.2–31.3	9.2 (5.3–13.1)
AIDS-free caregiver	17.8	12.3–23.2	14.6 (9.1–20.1)
Positive parenting & food sufficiency	19.1	11.6–26.6	13.3 (9.6–17.0)
Positive parenting & AIDS-free caregivers	14.3	9.2–19.4	18.1 (14.6–21.6)
Food sufficiency & AIDS-free caregivers	11.5	7.8–15.3	20.9 (18.0–23.8)
Positive parenting, food sufficiency & AIDS-free caregivers	9.0	5.6–12.4	23.4 (19.8–27.0)

## Results

Sociodemographic characteristics and prevalence of accelerators and SDG outcomes are shown in Table 1.

### Correlation matrix

Pairwise correlations between variables ranged from insignificant to *r = .*338 for physical and emotional abuse (Additional File 1: Supplement 1).

### Path analysis (Table 2 and Supplement 2)

The path model simultaneously containing all chosen SDG outcomes, hypothesized accelerator variables and covariates with correlated outcomes showed good model fit (*χ*^2^ = 258.387, df = 132, *p* < 001, CFI .967, TLI .914, RMSEA .017, WRMR .878). Table 2 displays the respective associations with the twelve outcomes. For example, reductions in physical abuse were predicted by positive parenting, food sufficiency, AIDS-free caregiver and parental good monitoring but not by teacher support nor free schooling.

### Gender invariance

Moderation of paths by sex was tested through path invariance analysis. The path invariance model, i.e. with the respective paths in the models for males and females set to equal each other, fit the data as well as the configural model: The Mplus DIFFTEST yielded Δ*χ*^2^(df) = 115.887(132), *p* = 0.840, meaning that moderation of paths by sex was not significant.

### Accelerator analysis

The Benjamini-Hochberg [25] procedure did not eliminate any predictors. Four development accelerators were identified, each positively associated with three or more SDG targets. Positive parenting was associated with less physical abuse, emotional abuse, substance use and suicidal ideation (SDGs 16.2, 3.4 and 3.5). High parental monitoring was associated with less physical abuse, emotional abuse, HIV risk behaviour, bullying, substance use and experiencing community violence (SDGs, 16.2, 5.61, 4.A, 3.5, 16.1). Having an AIDS-free caregiver was associated with less physical abuse, emotional abuse, experiencing community violence, suicidal ideation, bullying, witnessing domestic violence, TB symptomology and HIV risk behaviour (SDGs 16.2, 16.1, 3.4, 4.A, 5.2, 3.3, 5.61). Food sufficiency was associated with less physical abuse, emotional abuse, witnessing domestic violence, suicidal ideation, bullying, witnessing community violence, experiencing community violence and HIV risk behaviour (SDGs 16.2, 5.2, 3.4, 4.A, 16.1, 5.61). Free schooling and teacher support were not found to be accelerators (Table 2).

Correlations among outcomes in the path model, as compared with the pairwise correlations, proved to be quite substantial for physical and emotional abuse (*r =* 0.52), school dropout and physical abuse (*r =* 0.52), HIV risk behaviour and sexual abuse (*r =* 0.44) and substance use and HIV risk behaviour (*r =* 0.41). Other correlations were either weak or not statistically significant (Additional File 1: Supplement 2).

### Predicted percentage probabilities of accelerator impacts on SDG outcomes

**Fig. 2:**
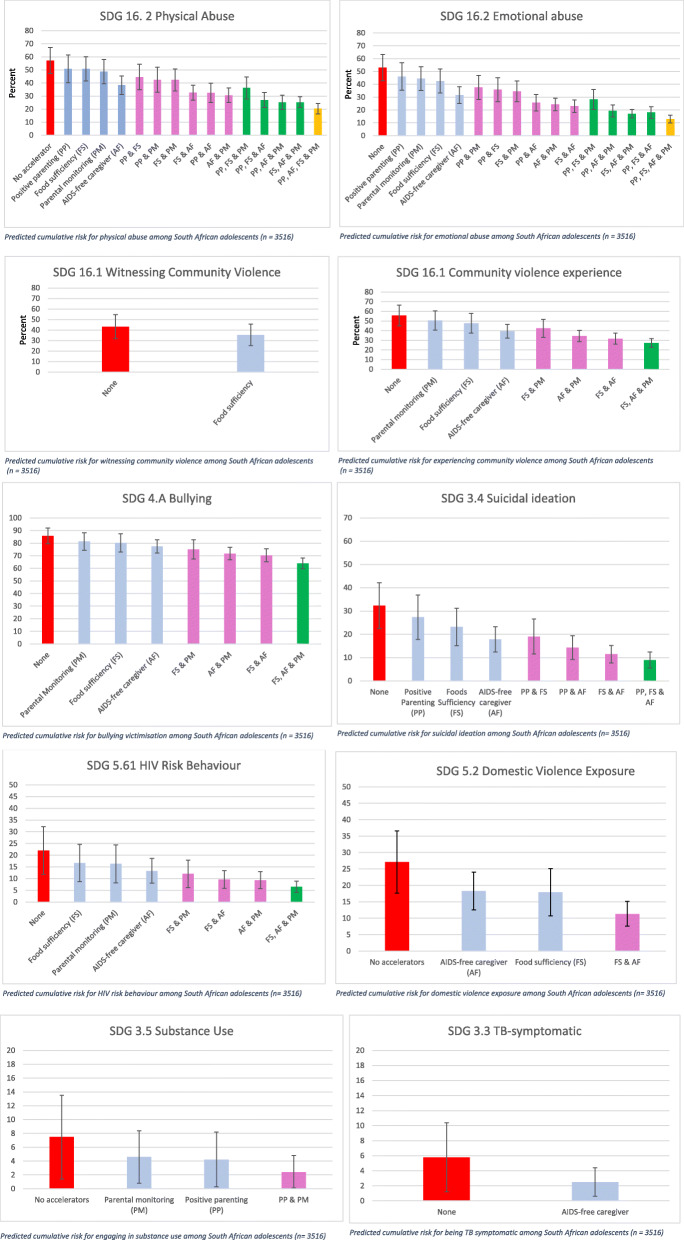
Percentage probability of SDG outcome occurrence in the presence of none, single and multiple accelerator combinations using marginal effects analysis

The predicted probability of physical abuse in this sample of South African adolescents was 57.2% (CI 47.2–67.1) with none of the accelerators present (Table 3, Fig. 2). This reduced to 50.9% (40.2–61.5) when in receipt of positive parenting, 50.9% (41.6–60.3) when food secure, 38.4% (31.3–45.5) when living with an AIDS-free caregiver and 48.7% (39.4–58.0) when experiencing good parental monitoring. Further reductions were observed for different combinations of two accelerators and greater for different combinations of three accelerators (see Table 3). Where all four accelerators were present, physical abuse exposure reduced to 25.2% (19.7–30.7). This is a percentage-point reduction of 33.2 (29.0–37.4) from no accelerators present.

The predicted probability of emotional abuse in this sample was 53.0% (CI 42.8–63.1) with none of the accelerators present (Table 3, Fig. 2). This reduced to 46.1% (35.4–56.7) when in receipt of positive parenting, 42.6% (33.2–51.9) when food secure, 31.7% (25.2–38.2) when living with an AIDS-free caregiver and 44.5% (35.2–53.7) when experiencing good parental monitoring. Further reductions were observed for different combinations of two accelerators and decreased further for different combinations of three accelerators (see Table 3). Where all four accelerators were present, physical abuse exposure reduced to 13.0% (10.0–16.1). This is a percentage-point reduction of 40.0% (35.9–44.1).

The predicted probability of bullying victimization in this sample was 85.7% (CI 79.4–91.9) with none of the accelerators present (Additional File 1: Table 3). This reduced to 80.2% (72.9–87.5) when food secure, 77.4% (72.1–82.6) when living with an AIDS-free caregiver and 81.3% (74.4–88.2) when experiencing good parental monitoring. Further reductions were observed for different combinations of two accelerators (Table 3, Fig. 2). Where all three accelerators were present, bullying victimization reduced to 64.0% (59.8–68.3). This is a percentage-point reduction of 21.7 (18.7–24.7).

The predicted probability of HIV risk behaviour in this sample was 22.0% (CI 11.8–32.2) with none of the accelerators present (Table 3). This reduced to 16.7% (8.8–24.7) when food secure, 13.3% (8.0–18.6) when living with an AIDS-free caregiver and 16.3% (8.2–24.4) when experiencing good parental monitoring. Further reductions were observed for different combinations of two accelerators (Table 3, Fig. 2). Where all three accelerators were present, HIV risk behaviour reduced to 6.5% (4.1–8.9). This is a percentage-point reduction of 15.5 (13.3–17.7).

The predicted probability of being TB symptomatic in this sample was 5.8% (CI 1.2–10.4) when no accelerators were present (Table 3). This reduced to 2.5% (0.6–4.4) when living with an AIDS-free caregiver. This is a percentage-point reduction of 3.3 (2.68–3.92) in TB symptomatic adolescents.

The predicted probability of witnessing domestic violence was 27.1% (17.6–36.6) in this sample. Exposure reduced to 17.9% (10.7–25.1) when food secure and 18.3% (12.6–24.1) when living with an AIDS-free caregiver. When both accelerators were present, witnessing domestic violence reduced to 11.3% (7.5–15.0). This is a percentage-point reduction of 15.8 (13.0–18.6) in domestic violence exposure.

The predicted probability of experiencing community violence was 55.7% (CI 45.0–66.4) when no accelerators were present (Table 3). This reduced to 47.6% (37.4–57.8) when food secure, 39.3% (32.2–46.4) when living with an AIDS-free caregiver and 50.5% (40.5–60.5) when experiencing good parental monitoring. This further reduced when combinations of two accelerators occurred (Table 3, Fig. 2). When all three accelerators were present, experience of community violence reduced to 27.2% (22.7–31.7). This is a percentage-point reduction of 28.5 (25.3–31.7) in community violence experience.

The predicted probability of witnessing community violence was 43.3% (CI 31.9–54.7) when no accelerators were present. This reduced to 35.3% (25.0–45.5) when food secure. This is a percentage-point reduction of 8.0 (3.7–12.3) in community violence exposure.

The predicted probability of engaging in substance use was 7.5% (CI 1.5–13.6) when no accelerators were present. This reduced to 4.2% (0.2–8.1) when in receipt of positive parenting and 4.6% (0.8–8.4) when experiencing parental monitoring. When both accelerators were present, substance use reduced to 2.4% (0.0–4.7). This is a percentage-point reduction of 5.1 (3.1–7.1) in substance use.

The predicted probability of suffering from suicidal ideation was 32.4% (CI 22.6–42.3) when no accelerators were present. This reduced to 27.4% (17.9–37.0) when receiving positive parenting, 23.2% (15.2–31.3) when food secure and 17.8% (12.3–23.2) when living with an AIDS-free caregiver. Further reductions were observed for different combinations of two accelerators (Table 3, Fig. 2). Suicide ideation reduced to 9% (5.6–12.4) when all three accelerators were present. This is a percentage-point reduction of 23.4 (19.9–27.0) in suicide ideation.

Across all outcomes where it was a significant accelerator, living with an AIDS-free caregiver was the accelerator contributing to the largest reduction in poor outcomes.

## Discussion

Aiming to achieve even a prioritized subset of the SDGs poses great challenges for lower- and middle-income countries and is even more challenging in countries with the highest HIV prevalence. Compared to upper-income countries, their governments have less tax revenue, and their populations include higher proportions of young people suffering a greater range of deprivations. Accelerators provide an essential opportunity, by identifying how particular interventions that might be undertaken, or particular circumstances that might be improved, can offer significant improvements on three or more SDG targets. Additionally, two or more accelerators may achieve enhanced additive impact on a particular outcome.

This study tested for accelerators among adolescents in especially highly HIV-prevalent and notably deprived communities in South Africa, and identified four accelerators which may aid government in reaching its SDGs in this population: living with an AIDS-free caregiver, food sufficiency, positive parenting, good parental monitoring. Each of these accelerators showed positive associations across at least three out of twelve SDG targets. Firstly, living with a caregiver who was not HIV-symptomatic was the accelerator with the most substantial association across SDG targets. This expands on evidence from South Africa which finds that living with an AIDS-unwell caregiver is associated with a large array of negative outcomes in adolescents [11] and puts children at heightened risk of violence exposure [27, 28]. Conversely, provision of universal access to treatment and testing and early initiation to antiretroviral medication in health systems, such as the 90-90-90 strategy, reduces the number of people with HIV symptomology [29]. It is poignant that the fundamental and essential need of keeping parents alive and healthy appears to be the most impactful accelerator tested in this research.

Secondly, food sufficiency was the accelerator with the next most substantial association across SDG targets. This amplifies a wide array of evidence from sub-Saharan Africa demonstrating that food sufficiency not only affects nutritional status, growth and weight, but is also strongly correlated with improved educational outcomes [30], improved mental health [31], reduced violence exposure and perpetration [32, 33] and reduced risk behaviours [33, 34]. Cash transfers and feeding programmes that reduce food insufficiency [35] will likely help governments achieve a number of SDG targets across multiple SDGs, and evidence from randomized trials [36] and other studies investigating accelerators seems to support this [10].

Thirdly, parenting in the form of very positive parenting and very good monitoring showed appreciable associations predominantly across SDG targets related to violence and mental health. This is in line with a growing body of evidence from small-scale evaluations on parenting programmes in sub-Saharan Africa, demonstrating their importance in child and adolescent development. In South Africa, they have been shown to increase positive parenting and parental monitoring which in turn reduce physical and emotional abuse [37], and show reductions in poor mental health expressed through behaviour problems and substance use [38, 39]. Thus far, parenting interventions have shown limited reach and are resource intense which can make their scale-up challenging in places where families experience structural issues. Evidence for the effectiveness of parenting interventions at scale is emerging through the COVID-19 parenting emergency response [40] which has to date reached 193 million people globally and which demonstrates some capacity for scale, with an evaluation in 11 countries showing increased parental engagement and play, more confidence in having positive relationships with their child, less physical abuse and less emotional abuse [41]. In addition, an evaluation of parenting programmes at scale in LMICs is under way [42] as are advances made in a new generation of digital parenting programmes which are yet to be deployed and evaluated but have scope for scale-up through their mechanism of delivery. It is important to note that poor parenting is rarely a choice, but rather an expression of structural issues faced by families such as financial stress, poor health and education systems or unsafe neighbourhoods [43]. Improvements in parenting will likely be seen when these structural issues, over which parents have limited control, are resolved [44].

In addition, this study identified additive effects on SDG targets, of accelerators in combination with each other. For example, food sufficiency was associated with improvements on seven SDG targets, but in combination with one or more of the other three accelerators, improvements on SDG targets were more substantial. This evidence supports that for adolescents to do well, they need to have three fundamental needs met: a parent who is alive and well, enough to eat and receive good parenting. In the context of very high HIV prevalence, none of these can be assumed and as such, these may need to be planned for to be provided together in order to achieve best possible outcomes for adolescents across multiple SDGs and their targets.

Fourthly, free schooling and teacher support were not considered accelerators as they each were not associated with three or more SDG outcomes. Each of these were individually associated with the SDG outcomes but those relationships were not sustained over and above the other pragmatic actions in the full model. These are important interventions in their own right, but in this context, for the purposes of this analysis, good parenting, AIDS-free caregiver and food sufficiency by themselves and in combination were found to be more important and future analyses should investigate this more. A future key area of investigation should focus on determinants of access to each accelerator and combinations of accelerators to allow governments to target populations lacking the presence of these accelerators in order to improve outcomes at the population level.

The path model also tested for moderation effects by sex, using the by-group analysis capability of Mplus. There was no significant difference. This is in contrast to other research from the area, using pooled cohorts, which finds significant differences in accelerators for boys and girls [45].

This study is subject to several limitations. First, the data in this study stem from black African adolescents in deprived settings within two provinces in South Africa. They are therefore not representative of South Africa as a whole. However, the study benefitted from in-sample variation with regard to location, sociodemographic characteristics, access to accelerators and SDG target outcomes. Second, the data from this study are now 10 years old which may limit the usefulness of the result. While the number of adolescents receiving school meals/free schooling and government cash transfers and access to antiretroviral has significantly increased in the years since the data were collected, the data serve as an important reminder of the importance of universal access to these types of interventions. In addition, there is evidence from recent accelerator studies in South Africa that receipt of cash transfers in areas with 95% coverage reduces risk for physical and emotional abuse and increases HIV care retention and school progression [10]. Further where state cash transfer coverage is near universal, randomized experiments have shown that impacts of cash transfers increase with the size of the transfer [46] and that additional payments on top of the government grant consistently reduce deprivation among girls in relation to violence, relationships as well as economically [47]. Third, accelerator provision was not based on receipt of interventions except for the free-school provision, but rather on real-world conditions existing in adolescent’s lives. Since the provision had to be received across both data collection points, it was not possible to determine whether the positive association of these provisions was due to more sustained or lifelong receipt, and whether provision of specific interventions could achieve the same outcomes. However, hypothesized accelerators were selected on the basis that they address behaviours or shortcomings which could be directly provided through real-world interventions, potentially underpinned by rigorous evidence from randomized experiments. Additionally, the findings do not establish causation. However, they do provide real-world evidence of provisions in which governments can invest, to help them achieve their SDG targets. Fourth, all measures employed in this study use self-report and are as such susceptible to social desirability bias. To mitigate measurement error, the study used validated and piloted measures which had been previously used with adolescents in South Africa and made use of specialist training for interviewers to encourage disclosure and trust in the research project. High retention rates at follow-up speak to the fact that adolescents were comfortable with participation. Fifth, while the study controlled for several potential sociodemographic confounders, the use of adolescent self-report precluded the collection of reliable data on parental income, mental health and educational status and future studies should make use of parent report to address this. Finally, the study conducted multiple-hypothesis testing which can result in erroneous inferences. However, this study corrected for multiple-hypothesis testing and also accounted for correlations among the multiple outcomes reducing the risk of such errors.

## Conclusion

This study demonstrates the potential of four accelerators, positive parenting and parental monitoring, food sufficiency and AIDS-free caregivers. These could translate to three real-world interventions: parenting programmes, cash transfers and early HIV treatment initiation and retention. With government commitment, these have shown high acceptability and cost-effectiveness. By combining them and expanding reach, governments in sub-Saharan Africa have the potential to assist their rapidly expanding adolescent populations to reach ten targets across four SDGs.

## Supplementary Information

**Additional File 1: Tables S1-S2**: **Table S1** - Pairwise correlation matrix. **Table S2** - Standardized correlation coefficients between SDG outcomes in the multivariate path model using the WLSMV estimator.

**Additional File 2:.** Stata and Mplus syntax used for the analysis

## Data Availability

The dataset generated and analysed during the current study are available from the corresponding author on reasonable request. The full dataset for the study is available here: Cluver, L. (2014). Young carers for AIDS-ill parents: social, health and educational impacts 2010-2013. [data collection]. UK Data Service. SN: 851277, 10.5255/UKDA-SN-851277.

## Abbreviations

SDG: Sustainable Development Goals
HIV: Human Immunodeficiency Virus
AIDS: Acquired Immunodeficiency Syndrome
UNDP: United Nations Development Programme
UNICEF: United Nations International Children’s Emergency Fund
CECV: Child Exposure to Community Violence Checklist
Mini-Kid: Mini international psychiatric interview for children and adolescents
APQ-SF: Alabama Parenting Questionnaire Short Form
WLSMV: Weighted least squares and variance adjusted
CFI: Comparative model fit
TLI: Tucker-Lewis index
RMSEA: Root mean square error of approximation
WRMR: Weighted root mean residual
