# Supplementary material for: Accelerating Sustainable Development Goals for South African adolescents from high HIV prevalence areas: a longitudinal path analysis
Source: BMC Med. 2021 Nov 11;19:263. doi: 10.1186/s12916-021-02137-8 (PMC8580740; doi:10.1186/s12916-021-02137-8)
Supplement: Supplementary file 1 — Additional File 1: Tables S1-S2: Table S1 - Pairwise correlation matrix. Table S2 - Standardized correlation coefficients between SDG outcomes in the multivariate path model using the WLSMV estimator. [file 12916_2021_2137_MOESM1_ESM.docx]

Supplement 1: Pairwise correlation matrix

|  | PA | EA | SA | BU | SRB | TB c | WDV | SchoolD | ECV | WCV | Subs | Suici | PP | HIV-/As | PM | FS | TS | FS | Age | PR | UR | Sex | IH |
| --- | --- | --- | --- | --- | --- | --- | --- | --- | --- | --- | --- | --- | --- | --- | --- | --- | --- | --- | --- | --- | --- | --- | --- |
| Physical Abuse | 1 |  |  |  |  |  |  |  |  |  |  |  |  |  |  |  |  |  |  |  |  |  |  |
| Emotional Abuse | 0.338*** | 1 |  |  |  |  |  |  |  |  |  |  |  |  |  |  |  |  |  |  |  |  |  |
| Sexual Abuse | 0.112*** | 0.170*** | 1 |  |  |  |  |  |  |  |  |  |  |  |  |  |  |  |  |  |  |  |  |
| Bullying | 0.124*** | 0.176*** | 0.084*** | 1 |  |  |  |  |  |  |  |  |  |  |  |  |  |  |  |  |  |  |  |
| Sexual Risk Behavior | -0.002 | 0.062*** | 0.204*** | 0.036* | 1 |  |  |  |  |  |  |  |  |  |  |  |  |  |  |  |  |  |  |
| TB symptomatic | 0.047** | 0.089*** | 0.065*** | 0.051** | 0.044* | 1 |  |  |  |  |  |  |  |  |  |  |  |  |  |  |  |  |  |
| Domestic Violence Witnessing | 0.134*** | 0.192*** | 0.059*** | 0.114*** | -0.002 | 0.074*** | 1 |  |  |  |  |  |  |  |  |  |  |  |  |  |  |  |  |
| School Dropout | -0.056** | -0.023 | 0.084*** | -0.042* | 0.180*** | -0.017 | 0.019 | 1 |  |  |  |  |  |  |  |  |  |  |  |  |  |  |  |
| Community Violence Experience | 0.061*** | 0.132*** | 0.007 | 0.142*** | 0.180*** | 0.017 | 0.134*** | 0.031 | 1 |  |  |  |  |  |  |  |  |  |  |  |  |  |  |
| Community Violence Exposure | -0.013 | 0.067*** | 0.019 | 0.109*** | 0.203*** | -0.033 | 0.074*** | 0.104*** | 0.334*** | 1 |  |  |  |  |  |  |  |  |  |  |  |  |  |
| Substance Use | 0.004 | 0.061*** | 0.046** | 0.027 | 0.314*** | 0.041* | 0.024 | 0.157*** | 0.144*** | 0.208*** | 1 |  |  |  |  |  |  |  |  |  |  |  |  |
| Suicide Ideation | 0.090*** | 0.213*** | 0.092*** | 0.157*** | 0.116*** | 0.056** | 0.146*** | 0.077*** | 0.186*** | 0.121*** | 0.035* | 1 |  |  |  |  |  |  |  |  |  |  |  |
| Positive Parenting | -0.031 | -0.084*** | -0.026 | -0.007 | -0.086*** | 0.002 | -0.034* | -0.056** | -0.121*** | -0.170*** | -0.103*** | -0.080*** | 1 |  |  |  |  |  |  |  |  |  |  |
| AIDS-free Caregiver | -0.105*** | -0.117*** | -0.028 | -0.054** | -0.035* | -0.064*** | -0.055** | -0.001 | -0.063*** | -0.012 | -0.005 | -0.088*** | 0.018 | 1 |  |  |  |  |  |  |  |  |  |
| Parental Monitoring | -0.034 | -0.082*** | -0.034* | -0.060*** | -0.164*** | 0.014 | -0.002 | -0.066*** | -0.112*** | -0.153*** | -0.157*** | -0.038* | 0.132*** | -0.010 | 1 |  |  |  |  |  |  |  |  |
| Food Sufficiency | -0.025 | -0.066*** | -0.023 | -0.047** | -0.041* | 0.027 | -0.067*** | -0.020 | -0.062*** | -0.084*** | -0.016 | -0.061*** | 0.052** | -0.000 | -0.013 | 1 |  |  |  |  |  |  |  |
| Teacher Support | -0.030 | -0.003 | -0.001 | -0.047** | -0.019 | 0.0018 | -0.034 | -0.088*** | 0.022 | 0.005 | -0.023 | -0.045** | 0.046** | 0.019 | 0.035* | 0.0204 | 1 |  |  |  |  |  |  |
| Free Schooling | 0.0303 | 0.013 | -0.023 | -0.011 | -0.068*** | -0.006 | -0.039* | -0.171*** | -0.004 | 0.063*** | -0.041* | -0.054** | -0.074*** | 0.001 | 0.005 | -0.035* | 0.049** | 1 |  |  |  |  |  |
| Age | -0.107*** | 0.044* | 0.120*** | -0.058*** | 0.281*** | -0.007 | -0.023 | 0.195*** | 0.069*** | 0.149*** | 0.221*** | 0.123*** | -0.108*** | 0.024 | -0.198*** | -0.0263 | -0.011 | -0.065*** | 1 |  |  |  |  |
| Province | 0.138*** | -0.028 | 0.082*** | -0.066*** | -0.169*** | 0.087*** | -0.041* | -0.070*** | -0.348*** | -0.545*** | -0.169*** | -0.093*** | 0.252*** | -0.012 | 0.175*** | 0.0773*** | 0.027 | -0.163*** | -0.082*** | 1 |  |  |  |
| Urban Location | 0.054** | 0.031 | 0.0422* | -0.113*** | 0.028 | -0.018 | -0.077*** | 0.0299 | -0.213*** | -0.020 | 0.0156 | -0.082*** | -0.041* | -0.019 | -0.032 | -0.0397* | -0.057*** | 0.340*** | 0.082*** | -0.008 | 1 |  |  |
| Sex | -0.021 | 0.049** | 0.057*** | -0.039* | -0.104*** | 0.028 | 0.052** | 0.034* | -0.006 | -0.026 | -0.078*** | 0.082*** | 0.012 | -0.033 | 0.081*** | -0.005 | 0.016 | 0.020 | 0.016 | -0.037* | 0.020 | 1 |  |
| Informal Housing | -0.083*** | -0.009 | -0.078*** | 0.057*** | 0.092*** | -0.041* | 0.021 | 0.046** | 0.181*** | 0.298*** | 0.053** | 0.045** | -0.172*** | -0.030 | -0.075*** | -0.028 | 0.007 | 0.117*** | 0.031 | -0.574*** | -0.066*** | 0.025 | 1 |

Supplement 2: Standardized correlation coefficients between SDG outcomes in the multivariate path model using the WLSMV estimator (n=3396)

|  | PA | EA | SA | BU | SRB | TB | WDV | SchoolD | ECV | WCV | Subs | Suici |
| --- | --- | --- | --- | --- | --- | --- | --- | --- | --- | --- | --- | --- |
| Physical Abuse | 1 |  |  |  |  |  |  |  |  |  |  |  |
| Emotional Abuse | 0.521* | 1 |  |  |  |  |  |  |  |  |  |  |
| Sexual Abuse | 0.248* | 0.342* | 1 |  |  |  |  |  |  |  |  |  |
| Bullying | 0.209* | 0.302* | 0.271* | 1 |  |  |  |  |  |  |  |  |
| HIV-Risk Behaviour | 0.077 | 0.095* | 0.444* | 0.069 | 1 |  |  |  |  |  |  |  |
| TB-Symptomatic | 0.063 | 0.219* | 0.182* | 0.151* | 0.215* | 1 |  |  |  |  |  |  |
| Witnessing Domestic Violence | 0.255* | 0.323* | 0.154* | 0.217* | -0.001 | 0.214* | 1 |  |  |  |  |  |
| School Dropout | 0.521* | -0.156* | 0.127 | -0.137* | 0.230* | -0.114 | 0.001 | 1 |  |  |  |  |
| Experiencing Community Violence | 0.216* | 0.196* | 0.067 | 0.179* | 0.254* | 0.136* | 0.208* | 0.004 | 1 |  |  |  |
| Witnessing Community Violence | 0.144* | 0.088* | 0.146* | 0.164* | 0.175* | 0.030 | 0.131* | 0.173* | 0.294* | 1 |  |  |
| Substance Use | 0.103* | 0.150* | 0.135* | 0.036 | 0.414* | 0.187* | 0.083 | 0.233* | 0.198* | 0.247* | 1 |  |
| Suicide Ideation | 0.202* | 0.329* | 0.151* | 0.331* | 0.151* | 0.162* | 0.232* | 0.065 | 0.244* | 0.129* | -0.027 | 1 |

*sig: p<.05
