# Supplementary material for: Accelerating Sustainable Development Goals for South African adolescents from high HIV prevalence areas: a longitudinal path analysis
Source: BMC Med. 2021 Nov 11;19:263. doi: 10.1186/s12916-021-02137-8 (PMC8580740; doi:10.1186/s12916-021-02137-8)
Supplement: Supplementary file 2 — Additional File 2:. Stata and Mplus syntax used for the analysis [file 12916_2021_2137_MOESM2_ESM.docx]

# Additional File 2: Syntax for analyses

Contents

[Additional File 2: Syntax for analyses 1](#_Toc78471921)

[Stage 1: Descriptive statistics in STATA 2](#_Toc78471922)

[Stage 2: Correlation matrix in STATA 3](#_Toc78471923)

[Stage 3: Multivariate (multiple-outcome) probit regressions with correlated outcomes in MPLUS 4](#_Toc78471924)

[Stage 4: Invariance testing 6](#_Toc78471925)

[1. Configural model 6](#_Toc78471926)

[2. Gender invariance test 10](#_Toc78471927)

[Stage 7: Predicted probabilities 14](#_Toc78471928)

## Stage 1: Descriptive statistics in STATA

tab ProvChild GenderChild, chi2 row col

ttest AgeofChildx, by(GenderChild)

mean AgeofChildx

tab PhysAbuseLYx GenderChild, chi2 row col

tab EmotAbuseLYx GenderChild, chi2 row col

tab SexAbuseLYx GenderChild, chi2 row col

tab Bullyx GenderChild, chi2 row col

tab SRHrisk1ormorex GenderChild, chi2 row col

tab TBtwosymx GenderChild, chi2 row col

tab DVArgumentsx GenderChild, chi2 row col

tab SchoolDropoutx GenderChild, chi2 row col

tab RobOrAssx GenderChild, chi2 row col

tab CommViolx GenderChild, chi2 row col

tab AlcOrDrugsx GenderChild, chi2 row col

tab SuicideIdeax GenderChild, chi2 row col

tab UrbanRuralx GenderChild, chi2 row col

tab Informalx GenderChild, chi2 row col

tab PhysAbuseT1LY GenderChild, chi2 row col

tab SexAbuseT1 GenderChild, chi2 row col

tab AnyEAbuseChildT1 GenderChild, chi2 row col

tab SRHrisk1ormore GenderChild, chi2 row col

tab Bully GenderChild, chi2 row col

tab TBtwosym GenderChild, chi2 row col

tab DVArguments GenderChild, chi2 row col

tab SchoolDropout GenderChild, chi2 row col

tab RobOrAss GenderChild, chi2 row col

tab CommViol GenderChild, chi2 row col

tab AlcOrDrugs GenderChild, chi2 row col

tab SuicideIdea GenderChild, chi2 row col

ttest AgeOfChild, by(GenderChild)

tab UrbanRural GenderChild, chi2 row col

tab Informal GenderChild, chi2 row col

tab PosParentT1andT2 NoHungerT1T2, chi2 row col

tab PosParentT1andT2 MonitoringAlwaysT1T2, chi2 row col

tab PosParentT1andT2 NoAIDSFamT1T2, chi2 row col

tab NoHungerT1T2 NoAIDSFamT1T2, chi2 row col

tab NoHungerT1T2 MonitoringAlwaysT1T2, chi2 row col

tab NoAIDSFamT1T2 MonitoringAlwaysT1T2, chi2 row col

## Stage 2: Correlation matrix in STATA

estpost correlate

PhysAbuseLYx EmotAbuseLYx SexAbuseLYx Bullyx SRHrisk1ormorex TBtwosymx DVArgumentsx SchoolDropoutx RobOrAssx CommViolx AlcOrDrugsx Suicidalx SuicideIdeax /// SDG outcomes

PosParentT1andT2 NoAIDSFamT1T2 MonitoringAlwaysT1T2 NoHungerT1T2

SSSTeachVeryT1T2 FreeSchoolBksFeesMealsT1T2 /// hypothesized accelerators

AgeofChild ProvChild UrbanRural GenderChild Informal, matrix

est store c1

esttab * using C:\Users\Franziska\Dropbox\CorrelationMatrix2.csv, unstack not noobs compress

## Stage 3: Multivariate (multiple-outcome) probit regressions with correlated outcomes in MPLUS

TITLE: Accelerator Analyses probit regressions

DATA: FILE IS "AcceleratorAnalysis.csv";

Variable:

NAMES ARE PALYx EALYx SALYx Bullyx SRHx TBx DVx

SchDropx Robx CommVx AlcDrx Suicix !SDG Outcomes

PAT1LY SAT1 EAT1 Bully SRHe TB DV

SchDr Rob CommV AlcDr Suici !baseline SDG Outcomes

PosP NoAIDS Monit NoHun SSSTeach FreeScho Formal !hypothesised accelerators

Age Prov UrbRu Gender; !covariates

MISSING ARE ALL (-9);

USEVARIABLES PALYx EALYx SALYx Bullyx SRHx TBx DVx

SchDropx Robx CommVx AlcDrx Suicix

PAT1LY SAT1 EAT1 Bully SRHe TB DV

SchDr Rob CommV AlcDr Suici

PosP NoAIDS Monit NoHun

SSSTeach FreeScho Formal

Age Prov UrbRu Gender;

CATEGORICAL ARE PALYx EALYx SALYx Bullyx SRHx TBx DVx

SchDropx Robx CommVx AlcDrx Suicix ;

ANALYSIS:

estimator=WLSMV;

PROCESSORS=4;

MODEL:

PALYx ON PosP NoAIDS Monit NoHun SSSTeach FreeScho

Formal Age Prov UrbRu Gender PAT1LY;

EALYx ON PosP NoAIDS Monit NoHun SSSTeach FreeScho

Formal Age Prov UrbRu Gender EAT1;

SALYx ON PosP NoAIDS Monit NoHun iSSSTeach FreeScho

Formal Age Prov UrbRu Gender SAT1;

Bullyx ON PosP NoAIDS Monit NoHun SSSTeach FreeScho

Formal Age Prov UrbRu Gender Bully;

SRHx ON PosP NoAIDS Monit NoHun SSSTeach FreeScho

Formal Age Prov UrbRu Gender SRHe;

TBx ON PosP NoAIDS Monit NoHun SSSTeach FreeScho

Formal Age Prov UrbRu Gender TB;

DVx ON PosP NoAIDS Monit NoHun SSSTeach FreeScho

Formal Age Prov UrbRu Gender DV;

SchDropx ON PosP NoAIDS Monit NoHun SSSTeach FreeScho

Formal Age Prov UrbRu Gender SchDr;

Robx ON PosP NoAIDS Monit NoHun SSSTeach FreeScho

Formal Age Prov UrbRu Gender Rob;

CommVx ON PosP NoAIDS Monit NoHun SSSTeach FreeScho

Formal Age Prov UrbRu Gender CommV;

AlcDrx ON PosP NoAIDS Monit NoHun SSSTeach FreeScho

Formal Age Prov UrbRu Gender AlcDr;

Suicix ON PosP NoAIDS Monit NoHun SSSTeach FreeScho

Formal Age Prov UrbRu Gender Suici;

!!!correlating outcomes

PALYx WITH EALYx-Suicix;

EALYx WITH SALYx-Suicix;

SALYx With Bullyx-Suicix;

Bullyx WITH SRHx-Suicix;

SRHx WITH TBx-Suicix;

TBx WITH DVx-Suicix;

DVx WITH SchDropx-Suicix;

SchDropx WITH Robx-Suicix;

Robx WITH CommVx-Suicix;

CommVx WITH AlcDrx-Suicix;

AlcDrx WITH Suicix;

OUTPUT:

SAMPSTAT STDYX CINTERVAL MODINDICES (10);

## Stage 4: Invariance testing

### Configural model

TITLE: Accelerator Analyses configural model

DATA: FILE IS "AcceleratorAnalysis_new2.csv";

Variable:

NAMES ARE P E S B H T D X R W A M !SDG Outcomes

PAT1LY SAT1 EAT1 Bully SRHe TB DV SchDr Rob CommV AlcDr Suici !SDG Outcomes at Baseline

PosP NoAIDS Monit NoHun SSSTeach FreeScho !hypothesised Accelerators

Informal Age Prov UrbRu Gender; !Covariates

MISSING ARE ALL (-9);

USEVARIABLES ARE P E S B H T D X R W A M !SDG Outcomes

PosP NoHun NoAIDS Monit SSSTeach FreeScho !hypothesised Accelerators

PAT1LY SAT1 EAT1 Bully SRHe TB DV SchDr Rob CommV AlcDr Suici !SDG Outcomes at Baseline

Age Prov UrbRu Informal; !Covariates

GROUPING = Gender (0=Boys, 1=Girls);

CATEGORICAL ARE P E S B H T D X R W A M;

ANALYSIS:

estimator=WLSMV;

PROCESSORS=4;

difftest = genderinv.dat;

MODEL:

P ON

PosP

NoHun

NoAIDS

Monit

SSSTeach

FreeScho

PAT1LY

Age

Prov

UrbRu

Informal;

E ON

PosP

NoHun

NoAIDS

Monit

SSSTeach

FreeScho

EAT1

Age

Prov

UrbRu

Informal;

S ON

PosP

NoHun

NoAIDS

Monit

SSSTeach

FreeScho

SAT1

Age

Prov

UrbRu

Informal;

B ON

PosP

NoHun

NoAIDS

Monit

SSSTeach

FreeScho

Bully

Age

Prov

UrbRu

Informal;

H ON

PosP

NoHun

NoAIDS

Monit

SSSTeach

FreeScho

SRHe

Age

Prov

UrbRu

Informal;

T ON

PosP

NoHun

NoAIDS

Monit

SSSTeach

FreeScho

TB

Age

Prov

UrbRu

Informal;

D ON

PosP

NoHun

NoAIDS

Monit

SSSTeach

FreeScho

DV

Age

Prov

UrbRu

Informal;

X ON

PosP

NoHun

NoAIDS

Monit

SSSTeach

FreeScho

SchDr

Age

Prov

UrbRu

Informal;

R ON

PosP

NoHun

NoAIDS

Monit

SSSTeach

FreeScho

Rob

Age

Prov

UrbRu

Informal;

W ON

PosP

NoHun

NoAIDS

Monit

SSSTeach

FreeScho

CommV

Age

Prov

UrbRu

Informal;

A ON

PosP

NoHun

NoAIDS

Monit

SSSTeach

FreeScho

AlcDr

Age

Prov

UrbRu

Informal;

M ON

PosP

NoHun

NoAIDS

Monit

SSSTeach

FreeScho

Suici

Age

Prov

UrbRu

Informal;

!NB Correlated outcomes

P WITH E-M;

E WITH S-M;

S WITH B-M;

B WITH H-M;

H WITH T-M;

T WITH D-M;

D WITH X-M;

X WITH R-M;

R WITH W-M;

W WITH A-M;

A WITH M;

savedata:

difftest is genderinv.dat;

OUTPUT:

STDYX CINTERVAL MODINDICES ; ! SAMPSTAT

### Gender invariance test

TITLE: Accelerator Analyses Invariance testing

DATA: FILE IS "AcceleratorAnalysis_new2.csv";

Variable:

NAMES ARE P E S B H T D X R W A M !SDG Outcomes

PAT1LY SAT1 EAT1 Bully SRHe TB DV SchDr Rob CommV AlcDr Suici !SDG Outcomes at Baseline

PosP NoAIDS Monit NoHun SSSTeach FreeScho !hypothesised Accelerators

Informal Age Prov UrbRu Gender; !Covariates

MISSING ARE ALL (-9);

USEVARIABLES ARE P E S B H T D X R W A M !SDG Outcomes

PosP NoHun NoAIDS Monit SSSTeach FreeScho !hypothesised Accelerators

PAT1LY SAT1 EAT1 Bully SRHe TB DV SchDr Rob CommV AlcDr Suici !SDG Outcomes at Baseline

Age Prov UrbRu Informal; !Covariates

GROUPING = Gender (0=Boys, 1=Girls);

CATEGORICAL ARE P E S B H T D X R W A M;

ANALYSIS:

estimator=WLSMV;

PROCESSORS=4;

difftest = genderinv.dat;

MODEL:

P ON

PosP (a1)

NoHun (a2)

NoAIDS (a3)

Monit (a4)

SSSTeach (a6)

FreeScho (a7)

PAT1LY (a9)

Age (a10)

Prov (a11)

UrbRu (a12)

Informal(a13);

E ON

PosP (b1)

NoHun (b2)

NoAIDS (b3)

Monit (b4)

SSSTeach (b6)

FreeScho (b7)

EAT1 (b9)

Age (b10)

Prov (b11)

UrbRu (b12)

Informal (b13);

S ON

PosP (c1)

NoHun (c2)

NoAIDS (c3)

Monit (c4)

SSSTeach (c6)

FreeScho (c7)

SAT1 (c9)

Age (c10)

Prov (c11)

UrbRu (c12)

Informal (c13);

B ON

PosP (d1)

NoHun (d2)

NoAIDS (d3)

Monit (d4)

SSSTeach (d6)

FreeScho (d7)

Bully (d9)

Age (d10)

Prov (d11)

UrbRu (d12)

Informal (d13);

H ON

PosP (e1)

NoHun (e2)

NoAIDS (e3)

Monit (e4)

SSSTeach (e6)

FreeScho (e7)

SRHe (e9)

Age (e10)

Prov (e11)

UrbRu (e12)

Informal (e13);

T ON

PosP (f1)

NoHun (f2)

NoAIDS (f3)

Monit (f4)

SSSTeach (f6)

FreeScho (f7)

TB (f9)

Age (f10)

Prov (f11)

UrbRu (f12)

Informal (f13);

D ON

PosP (g1)

NoHun (g2)

NoAIDS (g3)

Monit (g4)

SSSTeach (g6)

FreeScho (g7)

DV (g9)

Age (g10)

Prov (g11)

UrbRu (g12)

Informal (g13);

X ON

PosP (h1)

NoHun (h2)

NoAIDS (h3)

Monit (h4)

SSSTeach (h6)

FreeScho (h7)

SchDr (h9)

Age (h10)

Prov (h11)

UrbRu (h12)

Informal (h13);

R ON

PosP (i1)

NoHun (i2)

NoAIDS (i3)

Monit (i4)

SSSTeach (i6)

FreeScho (i7)

Rob (i9)

Age (i10)

Prov (i11)

UrbRu (i12)

Informal (i13);

W ON

PosP (j1)

NoHun (j2)

NoAIDS (j3)

Monit (j4)

SSSTeach (j6)

FreeScho (j7)

CommV (j9)

Age (j10)

Prov (j11)

UrbRu (j12)

Informal (j13);

A ON

PosP (k1)

NoHun (k2)

NoAIDS (k3)

Monit (k4)

SSSTeach (k6)

FreeScho (k7)

AlcDr (k9)

Age (k10)

Prov (k11)

UrbRu (k12)

Informal (k13);

M ON

PosP (L1)

NoHun (L2)

NoAIDS (L3)

Monit (L4)

SSSTeach (L6)

FreeScho (L7)

Suici (L9)

Age (L10)

Prov (L11)

UrbRu (L12)

Informal (L13);

!NB Correlated outcomes

P WITH E-M;

E WITH S-M;

S WITH B-M;

B WITH H-M;

H WITH T-M;

T WITH D-M;

D WITH X-M;

X WITH R-M;

R WITH W-M;

W WITH A-M;

A WITH M;

OUTPUT:

STDYX CINTERVAL MODINDICES ; ! SAMPSTAT

## Stage 7: Predicted probabilities

TITLE: Accelerator Analyses predicted probabilities

AS ABOVE

MODEL:

P ON

PosP (a1)

NoHun (a2)

NoAIDS (a3)

Monit (a4)

SSSTeach (a6)

FreeScho (a7)

PAT1LY (a9)

Age (a10)

Prov (a11)

UrbRu (a12)

Informal(a13)

Gender (a14);

[P$1] (a0);

E ON

PosP (b1)

NoHun (b2)

NoAIDS (b3)

Monit (b4)

SSSTeach (b6)

FreeScho (b7)

EAT1 (b9)

Age (b10)

Prov (b11)

UrbRu (b12)

Informal(b13)

Gender (b14);

[E$1] (b0);

S ON

PosP (c1)

NoHun (c2)

NoAIDS (c3)

Monit (c4)

SSSTeach (c6)

FreeScho (c7)

SAT1 (c9)

Age (c10)

Prov (c11)

UrbRu (c12)

Informal(c13)

Gender (c14);

[S$1] (c0);

B ON

PosP (d1)

NoHun (d2)

NoAIDS (d3)

Monit (d4)

SSSTeach (d6)

FreeScho (d7)

Bully (d9)

Age (d10)

Prov (d11)

UrbRu (d12)

Informal(d13)

Gender (d14);

[B$1] (d0);

H ON

PosP (e1)

NoHun (e2)

NoAIDS (e3)

Monit (e4)

SSSTeach (e6)

FreeScho (e7)

SRHe (e9)

Age (e10)

Prov (e11)

UrbRu (e12)

Informal(e13)

Gender (e14);

[H$1] (e0);

T ON

PosP (f1)

NoHun (f2)

NoAIDS (f3)

Monit (f4)

SSSTeach (f6)

FreeScho (f7)

TB (f9)

Age (f10)

Prov (f11)

UrbRu (f12)

Informal(f13)

Gender (f14);

[T$1] (f0);

D ON

PosP (g1)

NoHun (g2)

NoAIDS (g3)

Monit (g4)

SSSTeach (g6)

FreeScho (g7)

DV (g9)

Age (g10)

Prov (g11)

UrbRu (g12)

Informal(g13)

Gender (g14);

[D$1] (g0);

X ON

PosP (h1)

NoHun (h2)

NoAIDS (h3)

Monit (h4)

SSSTeach (h6)

FreeScho (h7)

SchDr (h9)

Age (h10)

Prov (h11)

UrbRu (h12)

Informal(h13)

Gender (h14);

[X$1] (h0);

R ON

PosP (i1)

NoHun (i2)

NoAIDS (i3)

Monit (i4)

SSSTeach (i6)

FreeScho (i7)

Rob (i9)

Age (i10)

Prov (i11)

UrbRu (i12)

Informal(i13)

Gender (i14);

[R$1] (i0);

W ON

PosP (j1)

NoHun (j2)

NoAIDS (j3)

Monit (j4)

SSSTeach (j6)

FreeScho (j7)

CommV (j9)

Age (j10)

Prov (j11)

UrbRu (j12)

Informal(j13)

Gender (j14);

[W$1] (j0);

A ON

PosP (k1)

NoHun (k2)

NoAIDS (k3)

Monit (k4)

SSSTeach (k6)

FreeScho (k7)

AlcDr (k9)

Age (k10)

Prov (k11)

UrbRu (k12)

Informal(k13)

Gender (k14);

[A$1] (k0);

M ON

PosP (L1)

NoHun (L2)

NoAIDS (L3)

Monit (L4)

SSSTeach (L6)

FreeScho (L7)

Suici (L9)

Age (L10)

Prov (L11)

UrbRu (L12)

Informal(L13)

Gender (L14);

[M$1] (L0);

!NB Correlated outcomes

P WITH E-M;

E WITH S-M;

S WITH B-M;

B WITH H-M;

H WITH T-M;

T WITH D-M;

D WITH X-M;

X WITH R-M;

R WITH W-M;

W WITH A-M;

A WITH M;

MODEL CONSTRAINT :

NEW (P000000 E000000 S000000 B000000 H000000 T000000

D000000 X000000 R000000 W000000 A000000 M000000

P200000 P020000 P002000 P000200 P000020 P000002

E200000 E020000 E002000 E000200 E000020 E000002

S200000 S020000 S002000 S000200 S000020 S000002

B200000 B020000 B002000 B000200 B000020 B000002

H200000 H020000 H002000 H000200 H000020 H000002

T200000 T020000 T002000 T000200 T000020 T000002

D200000 D020000 D002000 D000200 D000020 D000002

X200000 X020000 X002000 X000200 X000020 X000002

R200000 R020000 R002000 R000200 R000020 R000002

W200000 W020000 W002000 W000200 W000020 W000002

A200000 A020000 A002000 A000200 A000020 A000002

M200000 M020000 M002000 M000200 M000020 M000002

P222222 E222222 S222222 B222222 H222222 T222222

D222222 X222222 R222222 W222222 A222222 M222222

P222200 E222200 B022200 H022200 D022000 R022200

W020200 A200200 M222000

P220000 P222000 P022000 P202000 P202200 P002200 P022200

E220000 E222000 E022000 E202000 E202200 E002200 E022200

B022000 B002200 B020200

H022000 H002200 H020200

M220000 M202000 M022000

R022000 R020200 R002200

P200200 P020200 P220200

E200200 E020200 E220200);

P000000=phi(-a0+0*a1+0*a2+0*a3+0*a4+0*a6+0*a7

+a9*0.401+a10*14.671+a11*0.485+a12*0.494+a13*0.314+a14*0.566);

E000000=phi(-b0+0*b1+0*b2+0*b3+0*b4+0*b6+0*b7

+b9*0.037+b10*14.671+b11*0.485+b12*0.494+b13*0.314+b14*0.566);

S000000=phi(-c0+0*c1+0*c2+0*c3+0*c4+0*c6+0*c7

+c9*0.341+c10*14.671+c11*0.485+c12*0.494+c13*0.314+c14*0.566);

B000000=phi(-d0+0*d1+0*d2+0*d3+0*d4+0*d6+0*d7

+d9*0.744+d10*14.671+d11*0.485+d12*0.494+d13*0.314+d14*0.566);

H000000=phi(-e0+0*e1+0*e2+0*e3+0*e4+0*e6+0*e7

+e9*0.097+e10*14.671+e11*0.485+e12*0.494+e13*0.314+e14*0.566);

T000000=phi(-f0+0*f1+0*f2+0*f3+0*f4+0*f6+0*f7

+f9*0.105+f10*14.671+f11*0.485+f12*0.494+f13*0.314+f14*0.566);

D000000=phi(-g0+0*g1+0*g2+0*g3+0*g4+0*g6+0*g7

+g9*0.334+g10*14.671+g11*0.485+g12*0.494+g13*0.314+g14*0.566);

X000000=phi(-h0+0*h1+0*h2+0*h3+0*h4+0*h6+0*h7

+h9*0.016+h10*14.671+h11*0.485+h12*0.494+h13*0.314+h14*0.566);

R000000=phi(-i0+0*i1+0*i2+0*i3+0*i4+0*i6+0*i7

+i9*0.448+i10*14.671+i11*0.485+i12*0.494+i13*0.314+i14*0.566);

W000000=phi(-j0+0*j1+0*j2+0*j3+0*j4+0*j6+0*j7

+j9*0.413+j10*14.671+j11*0.485+j12*0.494+j13*0.314+j14*0.566);

A000000=phi(-k0+0*k1+0*k2+0*k3+0*k4+0*k6+0*k7

+k9*0.250+k10*14.671+k11*0.485+k12*0.494+k13*0.314+k14*0.566);

M000000=phi(-L0+0*L1+0*L2+0*L3+0*L4+0*L6+0*L7

+L9*0.165+L10*14.671+L11*0.485+L12*0.494+L13*0.314+L14*0.566);

P200000=phi(-a0+1*a1+0*a2+0*a3+0*a4+0*a6+0*a7

+a9*0.401+a10*14.671+a11*0.485+a12*0.494+a13*0.314+a14*0.566);

P020000=phi(-a0+0*a1+1*a2+0*a3+0*a4+0*a6+0*a7

+a9*0.401+a10*14.671+a11*0.485+a12*0.494+a13*0.314+a14*0.566);

P002000=phi(-a0+0*a1+0*a2+1*a3+0*a4+0*a6+0*a7

+a9*0.401+a10*14.671+a11*0.485+a12*0.494+a13*0.314+a14*0.566);

P000200=phi(-a0+0*a1+0*a2+0*a3+1*a4+0*a6+0*a7

+a9*0.401+a10*14.671+a11*0.485+a12*0.494+a13*0.314+a14*0.566);

P000020=phi(-a0+0*a1+0*a2+0*a3+0*a4+1*a6+0*a7

+a9*0.401+a10*14.671+a11*0.485+a12*0.494+a13*0.314+a14*0.566);

P000002=phi(-a0+0*a1+0*a2+0*a3+0*a4+0*a6+1*a7

+a9*0.401+a10*14.671+a11*0.485+a12*0.494+a13*0.314+a14*0.566);

E200000=phi(-b0+1*b1+0*b2+0*b3+0*b4+0*b6+0*b7

+b9*0.037+b10*14.671+b11*0.485+b12*0.494+b13*0.314+b14*0.566);

E020000=phi(-b0+0*b1+1*b2+0*b3+0*b4+0*b6+0*b7

+b9*0.037+b10*14.671+b11*0.485+b12*0.494+b13*0.314+b14*0.566);

E002000=phi(-b0+0*b1+0*b2+1*b3+0*b4+0*b6+0*b7

+b9*0.037+b10*14.671+b11*0.485+b12*0.494+b13*0.314+b14*0.566);

E000200=phi(-b0+0*b1+0*b2+0*b3+1*b4+0*b6+0*b7

+b9*0.037+b10*14.671+b11*0.485+b12*0.494+b13*0.314+b14*0.566);

E000020=phi(-b0+0*b1+0*b2+0*b3+0*b4+1*b6+0*b7

+b9*0.037+b10*14.671+b11*0.485+b12*0.494+b13*0.314+b14*0.566);

E000002=phi(-b0+0*b1+0*b2+0*b3+0*b4+0*b6+1*b7

+b9*0.037+b10*14.671+b11*0.485+b12*0.494+b13*0.314+b14*0.566);

S200000=phi(-c0+1*c1+0*c2+0*c3+0*c4+0*c6+0*c7

+c9*0.341+c10*14.671+c11*0.485+c12*0.494+c13*0.314+c14*0.566);

S020000=phi(-c0+0*c1+1*c2+0*c3+0*c4+0*c6+0*c7

+c9*0.341+c10*14.671+c11*0.485+c12*0.494+c13*0.314+c14*0.566);

S002000=phi(-c0+0*c1+0*c2+1*c3+0*c4+0*c6+0*c7

+c9*0.341+c10*14.671+c11*0.485+c12*0.494+c13*0.314+c14*0.566);

S000200=phi(-c0+0*c1+0*c2+0*c3+1*c4+0*c6+0*c7

+c9*0.341+c10*14.671+c11*0.485+c12*0.494+c13*0.314+c14*0.566);

S000020=phi(-c0+0*c1+0*c2+0*c3+0*c4+1*c6+0*c7

+c9*0.341+c10*14.671+c11*0.485+c12*0.494+c13*0.314+c14*0.566);

S000002=phi(-c0+0*c1+0*c2+0*c3+0*c4+0*c6+1*c7

+c9*0.341+c10*14.671+c11*0.485+c12*0.494+c13*0.314+c14*0.566);

B200000=phi(-d0+1*d1+0*d2+0*d3+0*d4+0*d6+0*d7

+d9*0.744+d10*14.671+d11*0.485+d12*0.494+d13*0.314+d14*0.566);

B020000=phi(-d0+0*d1+1*d2+0*d3+0*d4+0*d6+0*d7

+d9*0.744+d10*14.671+d11*0.485+d12*0.494+d13*0.314+d14*0.566);

B002000=phi(-d0+0*d1+0*d2+1*d3+0*d4+0*d6+0*d7

+d9*0.744+d10*14.671+d11*0.485+d12*0.494+d13*0.314+d14*0.566);

B000200=phi(-d0+0*d1+0*d2+0*d3+1*d4+0*d6+0*d7

+d9*0.744+d10*14.671+d11*0.485+d12*0.494+d13*0.314+d14*0.566);

B000020=phi(-d0+0*d1+0*d2+0*d3+0*d4+1*d6+0*d7

+d9*0.744+d10*14.671+d11*0.485+d12*0.494+d13*0.314+d14*0.566);

B000002=phi(-d0+0*d1+0*d2+0*d3+0*d4+0*d6+1*d7

+d9*0.744+d10*14.671+d11*0.485+d12*0.494+d13*0.314+d14*0.566);

H200000=phi(-e0+1*e1+0*e2+0*e3+0*e4+0*e6+0*e7

+e9*0.097+e10*14.671+e11*0.485+e12*0.494+e13*0.314+e14*0.566);

H020000=phi(-e0+0*e1+1*e2+0*e3+0*e4+0*e6+0*e7

+e9*0.097+e10*14.671+e11*0.485+e12*0.494+e13*0.314+e14*0.566);

H002000=phi(-e0+0*e1+0*e2+1*e3+0*e4+0*e6+0*e7

+e9*0.097+e10*14.671+e11*0.485+e12*0.494+e13*0.314+e14*0.566);

H000200=phi(-e0+0*e1+0*e2+0*e3+1*e4+0*e6+0*e7

+e9*0.097+e10*14.671+e11*0.485+e12*0.494+e13*0.314+e14*0.566);

H000020=phi(-e0+0*e1+0*e2+0*e3+0*e4+1*e6+0*e7

+e9*0.097+e10*14.671+e11*0.485+e12*0.494+e13*0.314+e14*0.566);

H000002=phi(-e0+0*e1+0*e2+0*e3+0*e4+0*e6+1*e7

+e9*0.097+e10*14.671+e11*0.485+e12*0.494+e13*0.314+e14*0.566);

T200000=phi(-f0+1*f1+0*f2+0*f3+0*f4+0*f6+0*f7

+f9*0.105+f10*14.671+f11*0.485+f12*0.494+f13*0.314+f14*0.566);

T020000=phi(-f0+0*f1+1*f2+0*f3+0*f4+0*f6+0*f7

+f9*0.105+f10*14.671+f11*0.485+f12*0.494+f13*0.314+f14*0.566);

T002000=phi(-f0+0*f1+0*f2+1*f3+0*f4+0*f6+0*f7

+f9*0.105+f10*14.671+f11*0.485+f12*0.494+f13*0.314+f14*0.566);

T000200=phi(-f0+0*f1+0*f2+0*f3+1*f4+0*f6+0*f7

+f9*0.105+f10*14.671+f11*0.485+f12*0.494+f13*0.314+f14*0.566);

T000020=phi(-f0+0*f1+0*f2+0*f3+0*f4+1*f6+0*f7

+f9*0.105+f10*14.671+f11*0.485+f12*0.494+f13*0.314+f14*0.566);

T000002=phi(-f0+0*f1+0*f2+0*f3+0*f4+0*f6+1*f7

+f9*0.105+f10*14.671+f11*0.485+f12*0.494+f13*0.314+f14*0.566);

D200000=phi(-g0+1*g1+0*g2+0*g3+0*g4+0*g6+0*g7

+g9*0.334+g10*14.671+g11*0.485+g12*0.494+g13*0.314+g14*0.566);

D020000=phi(-g0+0*g1+1*g2+0*g3+0*g4+0*g6+0*g7

+g9*0.334+g10*14.671+g11*0.485+g12*0.494+g13*0.314+g14*0.566);

D002000=phi(-g0+0*g1+0*g2+1*g3+0*g4+0*g6+0*g7

+g9*0.334+g10*14.671+g11*0.485+g12*0.494+g13*0.314+g14*0.566);

D000200=phi(-g0+0*g1+0*g2+0*g3+1*g4+0*g6+0*g7

+g9*0.334+g10*14.671+g11*0.485+g12*0.494+g13*0.314+g14*0.566);

D000020=phi(-g0+0*g1+0*g2+0*g3+0*g4+1*g6+0*g7

+g9*0.334+g10*14.671+g11*0.485+g12*0.494+g13*0.314+g14*0.566);

D000002=phi(-g0+0*g1+0*g2+0*g3+0*g4+0*g6+1*g7

+g9*0.334+g10*14.671+g11*0.485+g12*0.494+g13*0.314+g14*0.566);

X200000=phi(-h0+1*h1+0*h2+0*h3+0*h4+0*h6+0*h7

+h9*0.016+h10*14.671+h11*0.485+h12*0.494+h13*0.314+h14*0.566);

X020000=phi(-h0+0*h1+1*h2+0*h3+0*h4+0*h6+0*h7

+h9*0.016+h10*14.671+h11*0.485+h12*0.494+h13*0.314+h14*0.566);

X002000=phi(-h0+0*h1+0*h2+1*h3+0*h4+0*h6+0*h7

+h9*0.016+h10*14.671+h11*0.485+h12*0.494+h13*0.314+h14*0.566);

X000200=phi(-h0+0*h1+0*h2+0*h3+1*h4+0*h6+0*h7

+h9*0.016+h10*14.671+h11*0.485+h12*0.494+h13*0.314+h14*0.566);

X000020=phi(-h0+0*h1+0*h2+0*h3+0*h4+1*h6+0*h7

+h9*0.016+h10*14.671+h11*0.485+h12*0.494+h13*0.314+h14*0.566);

X000002=phi(-h0+0*h1+0*h2+0*h3+0*h4+0*h6+1*h7

+h9*0.016+h10*14.671+h11*0.485+h12*0.494+h13*0.314+h14*0.566);

R200000=phi(-i0+1*i1+0*i2+0*i3+0*i4+0*i6+0*i7

+i9*0.448+i10*14.671+i11*0.485+i12*0.494+i13*0.314+i14*0.566);

R020000=phi(-i0+0*i1+1*i2+0*i3+0*i4+0*i6+0*i7

+i9*0.448+i10*14.671+i11*0.485+i12*0.494+i13*0.314+i14*0.566);

R002000=phi(-i0+0*i1+0*i2+1*i3+0*i4+0*i6+0*i7

+i9*0.448+i10*14.671+i11*0.485+i12*0.494+i13*0.314+i14*0.566);

R000200=phi(-i0+0*i1+0*i2+0*i3+1*i4+0*i6+0*i7

+i9*0.448+i10*14.671+i11*0.485+i12*0.494+i13*0.314+i14*0.566);

R000020=phi(-i0+0*i1+0*i2+0*i3+0*i4+1*i6+0*i7

+i9*0.448+i10*14.671+i11*0.485+i12*0.494+i13*0.314+i14*0.566);

R000002=phi(-i0+0*i1+0*i2+0*i3+0*i4+0*i6+1*i7

+i9*0.448+i10*14.671+i11*0.485+i12*0.494+i13*0.314+i14*0.566);

W200000=phi(-j0+1*j1+0*j2+0*j3+0*j4+0*j6+0*j7

+j9*0.413+j10*14.671+j11*0.485+j12*0.494+j13*0.314+j14*0.566);

W020000=phi(-j0+0*j1+1*j2+0*j3+0*j4+0*j6+0*j7

+j9*0.413+j10*14.671+j11*0.485+j12*0.494+j13*0.314+j14*0.566);

W002000=phi(-j0+0*j1+0*j2+1*j3+0*j4+0*j6+0*j7

+j9*0.413+j10*14.671+j11*0.485+j12*0.494+j13*0.314+j14*0.566);

W000200=phi(-j0+0*j1+0*j2+0*j3+1*j4+0*j6+0*j7

+j9*0.413+j10*14.671+j11*0.485+j12*0.494+j13*0.314+j14*0.566);

W000020=phi(-j0+0*j1+0*j2+0*j3+0*j4+1*j6+0*j7

+j9*0.413+j10*14.671+j11*0.485+j12*0.494+j13*0.314+j14*0.566);

W000002=phi(-j0+0*j1+0*j2+0*j3+0*j4+0*j6+1*j7

+j9*0.413+j10*14.671+j11*0.485+j12*0.494+j13*0.314+j14*0.566);

A200000=phi(-k0+1*k1+0*k2+0*k3+0*k4+0*k6+0*k7

+k9*0.250+k10*14.671+k11*0.485+k12*0.494+k13*0.314+k14*0.566);

A020000=phi(-k0+0*k1+1*k2+0*k3+0*k4+0*k6+0*k7

+k9*0.250+k10*14.671+k11*0.485+k12*0.494+k13*0.314+k14*0.566);

A002000=phi(-k0+0*k1+0*k2+1*k3+0*k4+0*k6+0*k7

+k9*0.250+k10*14.671+k11*0.485+k12*0.494+k13*0.314+k14*0.566);

A000200=phi(-k0+0*k1+0*k2+0*k3+1*k4+0*k6+0*k7

+k9*0.250+k10*14.671+k11*0.485+k12*0.494+k13*0.314+k14*0.566);

A000020=phi(-k0+0*k1+0*k2+0*k3+0*k4+1*k6+0*k7

+k9*0.250+k10*14.671+k11*0.485+k12*0.494+k13*0.314+k14*0.566);

A000002=phi(-k0+0*k1+0*k2+0*k3+0*k4+0*k6+1*k7

+k9*0.250+k10*14.671+k11*0.485+k12*0.494+k13*0.314+k14*0.566);

M200000=phi(-L0+1*L1+0*L2+0*L3+0*L4+0*L6+0*L7

+L9*0.165+L10*14.671+L11*0.485+L12*0.494+L13*0.314+L14*0.566);

M020000=phi(-L0+0*L1+1*L2+0*L3+0*L4+0*L6+0*L7

+L9*0.165+L10*14.671+L11*0.485+L12*0.494+L13*0.314+L14*0.566);

M002000=phi(-L0+0*L1+0*L2+1*L3+0*L4+0*L6+0*L7

+L9*0.165+L10*14.671+L11*0.485+L12*0.494+L13*0.314+L14*0.566);

M000200=phi(-L0+0*L1+0*L2+0*L3+1*L4+0*L6+0*L7

+L9*0.165+L10*14.671+L11*0.485+L12*0.494+L13*0.314+L14*0.566);

M000020=phi(-L0+0*L1+0*L2+0*L3+0*L4+1*L6+0*L7

+L9*0.165+L10*14.671+L11*0.485+L12*0.494+L13*0.314+L14*0.566);

M000002=phi(-L0+0*L1+0*L2+0*L3+0*L4+0*L6+1*L7

+L9*0.165+L10*14.671+L11*0.485+L12*0.494+L13*0.314+L14*0.566);

P222222=phi(-a0+1*a1+1*a2+1*a3+1*a4+1*a6+1*a7

+a9*0.401+a10*14.671+a11*0.485+a12*0.494+a13*0.314+a14*0.566);

E222222=phi(-b0+1*b1+1*b2+1*b3+1*b4+1*b6+1*b7

+b9*0.037+b10*14.671+b11*0.485+b12*0.494+b13*0.314+b14*0.566);

S222222=phi(-c0+1*c1+1*c2+1*c3+1*c4+1*c6+1*c7

+c9*0.341+c10*14.671+c11*0.485+c12*0.494+c13*0.314+c14*0.566);

B222222=phi(-d0+1*d1+1*d2+1*d3+1*d4+1*d6+1*d7

+d9*0.744+d10*14.671+d11*0.485+d12*0.494+d13*0.314+d14*0.566);

H222222=phi(-e0+1*e1+1*e2+1*e3+1*e4+1*e6+1*e7

+e9*0.097+e10*14.671+e11*0.485+e12*0.494+e13*0.314+e14*0.566);

T222222=phi(-f0+1*f1+1*f2+1*f3+1*f4+1*f6+1*f7

+f9*0.105+f10*14.671+f11*0.485+f12*0.494+f13*0.314+f14*0.566);

D222222=phi(-g0+1*g1+1*g2+1*g3+1*g4+1*g6+1*g7

+g9*0.334+g10*14.671+g11*0.485+g12*0.494+g13*0.314+g14*0.566);

X222222=phi(-h0+1*h1+1*h2+1*h3+1*h4+1*h6+1*h7

+h9*0.016+h10*14.671+h11*0.485+h12*0.494+h13*0.314+h14*0.566);

R222222=phi(-i0+1*i1+1*i2+1*i3+1*i4+1*i6+1*i7

+i9*0.448+i10*14.671+i11*0.485+i12*0.494+i13*0.314+i14*0.566);

W222222=phi(-j0+1*j1+1*j2+1*j3+1*j4+1*j6+1*j7

+j9*0.413+j10*14.671+j11*0.485+j12*0.494+j13*0.314+j14*0.566);

A222222=phi(-k0+1*k1+1*k2+1*k3+1*k4+1*k6+1*k7

+k9*0.250+k10*14.671+k11*0.485+k12*0.494+k13*0.314+k14*0.566);

M222222=phi(-L0+1*L1+1*L2+1*L3+1*L4+1*L6+1*L7

+L9*0.165+L10*14.671+L11*0.485+L12*0.494+L13*0.314+L14*0.566);

P222200=phi(-a0+1*a1+1*a2+1*a3+1*a4+0*a6+0*a7

+a9*0.401+a10*14.671+a11*0.485+a12*0.494+a13*0.314+a14*0.566);

E222200=phi(-b0+1*b1+1*b2+1*b3+1*b4+0*b6+0*b7

+b9*0.037+b10*14.671+b11*0.485+b12*0.494+b13*0.314+b14*0.566);

B022200=phi(-d0+0*d1+1*d2+1*d3+1*d4+0*d6+0*d7

+d9*0.744+d10*14.671+d11*0.485+d12*0.494+d13*0.314+d14*0.566);

H022200=phi(-e0+0*e1+1*e2+1*e3+1*e4+0*e6+0*e7 !error here, must take new results

+e9*0.097+e10*14.671+e11*0.485+e12*0.494+e13*0.314+e14*0.566);

D022000=phi(-g0+0*g1+1*g2+1*g3+0*g4+0*g6+0*g7

+g9*0.334+g10*14.671+g11*0.485+g12*0.494+g13*0.314+g14*0.566);

W020200=phi(-j0+0*j1+1*j2+0*j3+1*j4+0*j6+0*j7

+j9*0.413+j10*14.671+j11*0.485+j12*0.494+j13*0.314+j14*0.566);

A200200=phi(-k0+1*k1+0*k2+0*k3+1*k4+0*k6+0*k7

+k9*0.250+k10*14.671+k11*0.485+k12*0.494+k13*0.314+k14*0.566);

M222000=phi(-L0+1*L1+1*L2+1*L3+0*L4+0*L6+0*L7

+L9*0.165+L10*14.671+L11*0.485+L12*0.494+L13*0.314+L14*0.566);

R022200=phi(-i0+0*i1+1*i2+1*i3+1*i4+0*i6+0*i7

+i9*0.448+i10*14.671+i11*0.485+i12*0.494+i13*0.314+i14*0.566);

!different combinations of accelerators

P220000=phi(-a0+1*a1+1*a2+0*a3+0*a4+0*a6+0*a7

+a9*0.401+a10*14.671+a11*0.485+a12*0.494+a13*0.314+a14*0.566);

P222000=phi(-a0+1*a1+1*a2+1*a3+0*a4+0*a6+0*a7

+a9*0.401+a10*14.671+a11*0.485+a12*0.494+a13*0.314+a14*0.566);

P022000=phi(-a0+0*a1+1*a2+1*a3+0*a4+0*a6+0*a7

+a9*0.401+a10*14.671+a11*0.485+a12*0.494+a13*0.314+a14*0.566);

P202000=phi(-a0+1*a1+0*a2+1*a3+0*a4+0*a6+0*a7

+a9*0.401+a10*14.671+a11*0.485+a12*0.494+a13*0.314+a14*0.566);

P202200=phi(-a0+1*a1+0*a2+1*a3+1*a4+0*a6+0*a7

+a9*0.401+a10*14.671+a11*0.485+a12*0.494+a13*0.314+a14*0.566);

P002200=phi(-a0+0*a1+0*a2+1*a3+1*a4+0*a6+0*a7

+a9*0.401+a10*14.671+a11*0.485+a12*0.494+a13*0.314+a14*0.566);

P022200=phi(-a0+0*a1+1*a2+1*a3+1*a4+0*a6+0*a7

+a9*0.401+a10*14.671+a11*0.485+a12*0.494+a13*0.314+a14*0.566);

E220000=phi(-b0+1*b1+1*b2+0*b3+0*b4+0*b6+0*b7

+b9*0.037+b10*14.671+b11*0.485+b12*0.494+b13*0.314+b14*0.566);

E222000=phi(-b0+1*b1+1*b2+1*b3+0*b4+0*b6+0*b7

+b9*0.037+b10*14.671+b11*0.485+b12*0.494+b13*0.314+b14*0.566);

E022000=phi(-b0+0*b1+1*b2+1*b3+0*b4+0*b6+0*b7

+b9*0.037+b10*14.671+b11*0.485+b12*0.494+b13*0.314+b14*0.566);

E202000=phi(-b0+1*b1+0*b2+1*b3+0*b4+0*b6+0*b7

+b9*0.037+b10*14.671+b11*0.485+b12*0.494+b13*0.314+b14*0.566);

E202200=phi(-b0+1*b1+0*b2+1*b3+1*b4+0*b6+0*b7

+b9*0.037+b10*14.671+b11*0.485+b12*0.494+b13*0.314+b14*0.566);

E002200=phi(-b0+0*b1+0*b2+1*b3+1*b4+0*b6+0*b7

+b9*0.037+b10*14.671+b11*0.485+b12*0.494+b13*0.314+b14*0.566);

E022200=phi(-b0+0*b1+1*b2+1*b3+1*b4+0*b6+0*b7

+b9*0.037+b10*14.671+b11*0.485+b12*0.494+b13*0.314+b14*0.566);

B022000=phi(-d0+0*d1+1*d2+1*d3+0*d4+0*d6+0*d7

+d9*0.744+d10*14.671+d11*0.485+d12*0.494+d13*0.314+d14*0.566);

B002200=phi(-d0+0*d1+0*d2+1*d3+1*d4+0*d6+0*d7

+d9*0.744+d10*14.671+d11*0.485+d12*0.494+d13*0.314+d14*0.566);

B020200=phi(-d0+0*d1+1*d2+0*d3+1*d4+0*d6+0*d7

+d9*0.744+d10*14.671+d11*0.485+d12*0.494+d13*0.314+d14*0.566);

H022000=phi(-e0+0*e1+1*e2+1*e3+0*e4+0*e6+0*e7

+e9*0.097+e10*14.671+e11*0.485+e12*0.494+e13*0.314+e14*0.566);

H002200=phi(-e0+0*e1+0*e2+1*e3+1*e4+0*e6+0*e7

+e9*0.097+e10*14.671+e11*0.485+e12*0.494+e13*0.314+e14*0.566);

H020200=phi(-e0+0*e1+1*e2+0*e3+1*e4+0*e6+0*e7

+e9*0.097+e10*14.671+e11*0.485+e12*0.494+e13*0.314+e14*0.566);

M220000=phi(-L0+1*L1+1*L2+0*L3+0*L4+0*L6+0*L7

+L9*0.165+L10*14.671+L11*0.485+L12*0.494+L13*0.314+L14*0.566);

M202000=phi(-L0+1*L1+0*L2+1*L3+0*L4+0*L6+0*L7

+L9*0.165+L10*14.671+L11*0.485+L12*0.494+L13*0.314+L14*0.566);

M022000=phi(-L0+0*L1+1*L2+1*L3+0*L4+0*L6+0*L7

+L9*0.165+L10*14.671+L11*0.485+L12*0.494+L13*0.314+L14*0.566);

R022000=phi(-i0+0*i1+1*i2+1*i3+0*i4+0*i6+0*i7

+i9*0.448+i10*14.671+i11*0.485+i12*0.494+i13*0.314+i14*0.566);

R020200=phi(-i0+0*i1+1*i2+0*i3+1*i4+0*i6+0*i7

+i9*0.448+i10*14.671+i11*0.485+i12*0.494+i13*0.314+i14*0.566);

R002200=phi(-i0+0*i1+0*i2+1*i3+1*i4+0*i6+0*i7

+i9*0.448+i10*14.671+i11*0.485+i12*0.494+i13*0.314+i14*0.566);

P200200=phi(-a0+1*a1+0*a2+0*a3+1*a4+0*a6+0*a7

+a9*0.401+a10*14.671+a11*0.485+a12*0.494+a13*0.314+a14*0.566);

P020200=phi(-a0+0*a1+1*a2+0*a3+1*a4+0*a6+0*a7

+a9*0.401+a10*14.671+a11*0.485+a12*0.494+a13*0.314+a14*0.566);

P220200=phi(-a0+1*a1+1*a2+0*a3+1*a4+0*a6+0*a7

+a9*0.401+a10*14.671+a11*0.485+a12*0.494+a13*0.314+a14*0.566);

E200200=phi(-b0+1*b1+0*b2+0*b3+1*b4+0*b6+0*b7

+b9*0.037+b10*14.671+b11*0.485+b12*0.494+b13*0.314+b14*0.566);

E020200=phi(-b0+0*b1+1*b2+0*b3+1*b4+0*b6+0*b7

+b9*0.037+b10*14.671+b11*0.485+b12*0.494+b13*0.314+b14*0.566);

E220200=phi(-b0+1*b1+1*b2+0*b3+1*b4+0*b6+0*b7

+b9*0.037+b10*14.671+b11*0.485+b12*0.494+b13*0.314+b14*0.566);

OUTPUT:

STDYX CINTERVAL ; ! SAMPSTAT
